# Imaging of Recombination Rates and Lifetime in Perovskite Thin Film Processing

**DOI:** 10.1002/smtd.202402119

**Published:** 2025-02-16

**Authors:** Benjamin Hacene, Nils W. Rosemann, Julie Roger, Xuzheng Liu, Daniel O. Baumann, Ronja Pappenberger, Mohammad Gholipoor, Hannah Racky, Paul Fassl, Ian A. Howard, Ulrich W. Paetzold

**Affiliations:** ^1^ Light Technology Institute (LTI) Karlsruhe Institute of Technology (KIT) Engesserstrasse 13 76131 Karlsruhe Germany; ^2^ Institute of Microstructure Technology (IMT) Karlsruhe Institute of Technology (KIT), Hermann‐von‐Helmholtz‐Platz 1 76344 Eggenstein‐Leopoldshafen Germany

**Keywords:** imaging, lifetime, perovskite, photoluminescence, recombination rates

## Abstract

Large‐scale fabrication and optimization of high‐quality polycrystalline perovskite thin films present significant challenges in scientific research and industry. Shifting from single‐spot measurements to imaging techniques facilitates the transition from laboratory‐scale to large‐scale processing. While single‐spot photoluminescence (PL) methods provide high‐depth insights into local optoelectronic characteristics, they are insufficient for assessing reliable information on homogeneity and spatial characteristics. Currently, no PL‐based imaging method delivers a comparable level of information depth on charge carrier dynamics to single‐spot PL methods. In response, this work introduces a non‐invasive imaging technique based on double‐pulse excitation. Varying the time delay between the pulses gives rise to spatial information on relative photoluminescence quantum yield (rPLQY) in thin films, yielding fundamental optoelectronic characteristics such as external radiative and effective non‐radiative recombination rates and charge‐carrier lifetime. Compared to traditional PL‐imaging and *k*‐imaging demonstrates the superiority of rPLQY‐imaging in revealing charge carrier dynamics. This technique proves applicable across various sample configurations, irrespective of the presence of electrodes and charge transport layers. In addition, the method can estimate surface recombination velocity and variations in escape and parasitic absorption probabilities. Overall, the rPLQY‐imaging method emerges as a valuable tool for scientific research aimed at characterizing and optimizing large‐area perovskite thin films.

## Graphical abstract

Achieving consistent, homogeneous layer quality in perovskite thin films is a critical challenge for large‐scale processing. This study introduces relative photoluminescence quantum yield (rPLQY)‐imaging, a novel, non‐invasive imaging technique that enables spatially resolved characterization of fundamental optoelectronic characteristics. This method provides valuable insights into recombination processes and enhances optimization potential across various sample configurations.

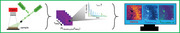

## 1 Introduction

**Figure 1:**
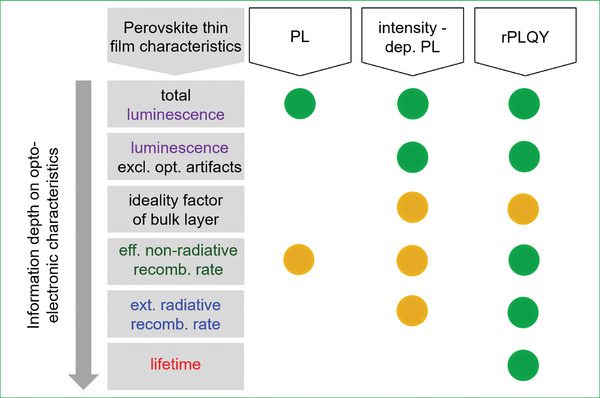
Schematic comparison of traditional photoluminescence (PL)‐, intensity‐dependent PL, and relative photoluminescence quantum yield (rPLQY)‐imaging for their respective information depth with regard to several characteristic parameters. The yellow and green circles indicate qualitative and quantitative information on the characteristics delivered by the respective imaging method.

Perovskite thin film photovoltaics sustain tremendous attention due to their excellent power conversion efficiencies (PCEs), tunable bandgaps, and cost‐effective fabrication methods.^[^ ^1^ ^]^ Single‐junction perovskite solar cells achieve PCEs exceeding 26%,^[^ ^2^ ^]^ rendering them competitive with the established silicon‐based technology.^[^ ^3^ ^]^ Moreover, silicon‐perovskite tandem solar cells (TSCs) demonstrate remarkable PCEs surpassing 34% on a laboratory scale,^[^ ^4^ ^]^ highlighting the enormous potential of the technology. Mitigating non‐radiative recombination processes, detrimental to the open‐circuit voltage (*V*_OC_), constitutes a promising approach to further improve the device's performance.^[^ ^5^ ^]^ Achieving such improvements requires a deeper understanding of the underlying physics of charge carrier transport, recombination dynamics, and material degradation mechanisms.^[^ ^6^ ^]^ Photoluminescence (PL) based characterization techniques are non‐invasive and provide insights into excited charge carriers' radiative and non‐radiative recombination processes.^[^ ^7^, ^8^, ^9^ ^]^ These methods enable an understanding of recombination dynamics, which are crucial for sample characterization and optimization.^[^ ^10^ ^]^ Moreover, PL‐based imaging techniques provide access to spatial variations within the sample, a crucial attribute for large‐scale thin film characterization. In contrast to spin‐coated samples, large‐scale solution‐processed perovskite thin films deposited via blade or slot‐die coating often exhibit increased inhomogeneity and defect density.^[^ ^11^ ^]^ Scalable characterization methods are therefore essential for achieving a comprehensive evaluation of thin film quality and homogeneity. Established techniques such as traditional PL and intensity‐dependent PL imaging differ in their routine of sample excitation, leading to different degrees of information depth on optoelectronic characteristics. However, both methods provide only qualitative information on recombination rates^[^ ^11^ ^]^ (see **Figure** **1**). In this work, we introduce relative photoluminescence quantum yield (rPLQY) imaging, a novel and complementary PL‐based imaging methodology that is capable of directly determining the rate constants. To demonstrate the advance of rPLQY‐imaging compared to traditional and intensity‐dependent PL imaging, we briefly review the methods, as detailed below:

1. *Traditional PL imaging* typically involves exciting the sample with a predefined intensity and wavelength, followed by image acquisition using a scientific camera. A long pass filter effectively blocks the excitation light, ensuring accurate detection of emitted PL signals. The measurement can be affected by various sources, such as stray light, reflections, or uneven illumination, which can compromise the result's reliability.^[^ ^12^ ^]^ Additionally, different excitation conditions are reported in the literature, which complicates comparisons between different setups as PL signals are typically measured in arbitrary units. Moreover, only qualitative assessments of non‐radiative recombination can typically be made based on such data.^[^ ^13^ ^]^ Higher PL intensity is often associated with a lower extent of non‐radiative recombination rates, suggesting better film quality. However, interpreting the results can be challenging due to additional factors, such as photon in‐ and outcoupling at the film surface.^[^ ^14^ ^]^
2. *Intensity‐dependent PL imaging* provides further insight into recombination kinetics, as PL intensity typically follows a power law relationship PL∝*I^k^* with the parameter *k* and illumination intensity *I*. When free charge carriers govern the photon emission, the parameter *k* ranges between 1 and 2. A *k*‐value of 1 signifies a dominant radiative recombination path or non‐radiative surface recombination, whereas a *k*‐value of 2 indicates a predominance of non‐radiative channels. Since the *k*‐parameter does not provide absolute recombination rate values, interpreting variations in *k* is ambiguous. Consequently, *k*‐values offer qualitative insights into the effective non‐radiative and external radiative rate constants *k*_non‐radiative, eff_, and *k*_radiative, ext_.^[^ ^15^, ^16^, ^17^ ^]^ The *k*‐imaging technique provides quantitative results comparable to other experimental techniques. Moreover, it shows robustness against inhomogeneous illumination or reflection effects, enhancing information depth and reliability in contrast to conventional PL imaging.^[^ ^11^ ^]^
3. *Relative photoluminescence quantum yield imaging*, introduced in this work, is based on double‐pulse (DP) excitation with variable delay time between the pulses and directly provides the quantitative values of both rate constants, *k*_non‐radiative, eff_ and *k*_radiative, ext_. Since PL‐imaging offers qualitative information about *k*_non‐radiative, eff_ and k‐imaging about *k*_non‐radiative, eff_ and *k*_radiative, ext_, the direct quantification of recombination rates in rPLQY‐imaging establishes it as the most reliable method by unambiguously assessing the quality of a sample and showing the highest information depth among these three PL‐based imaging techniques (see Figure 1).

To date, no concept has been established to perform rPLQY‐imaging. In response, this study introduces the rPLQY‐imaging methodology and the fundamental physics underlying this technique. We validate its reliability by comparing results with the established time‐correlated single photon counting (TCSPC) technique. We highlight the advantages of obtaining spatially resolved quantitative values of rate constants for scientific applications. Numerical experimentation illustrates the broad range of compatible sample configurations and outlines inherent limitations. Furthermore, application on strategically designed samples gives rise to an estimation of surface recombination velocity and variation in escape and parasitic absorption probability. Overall, this study showcases the suitability of this methodology for scientific purposes and future integration in industrial processes.

## 2 Results

### 2.1 Methodology

**Figure 2:**
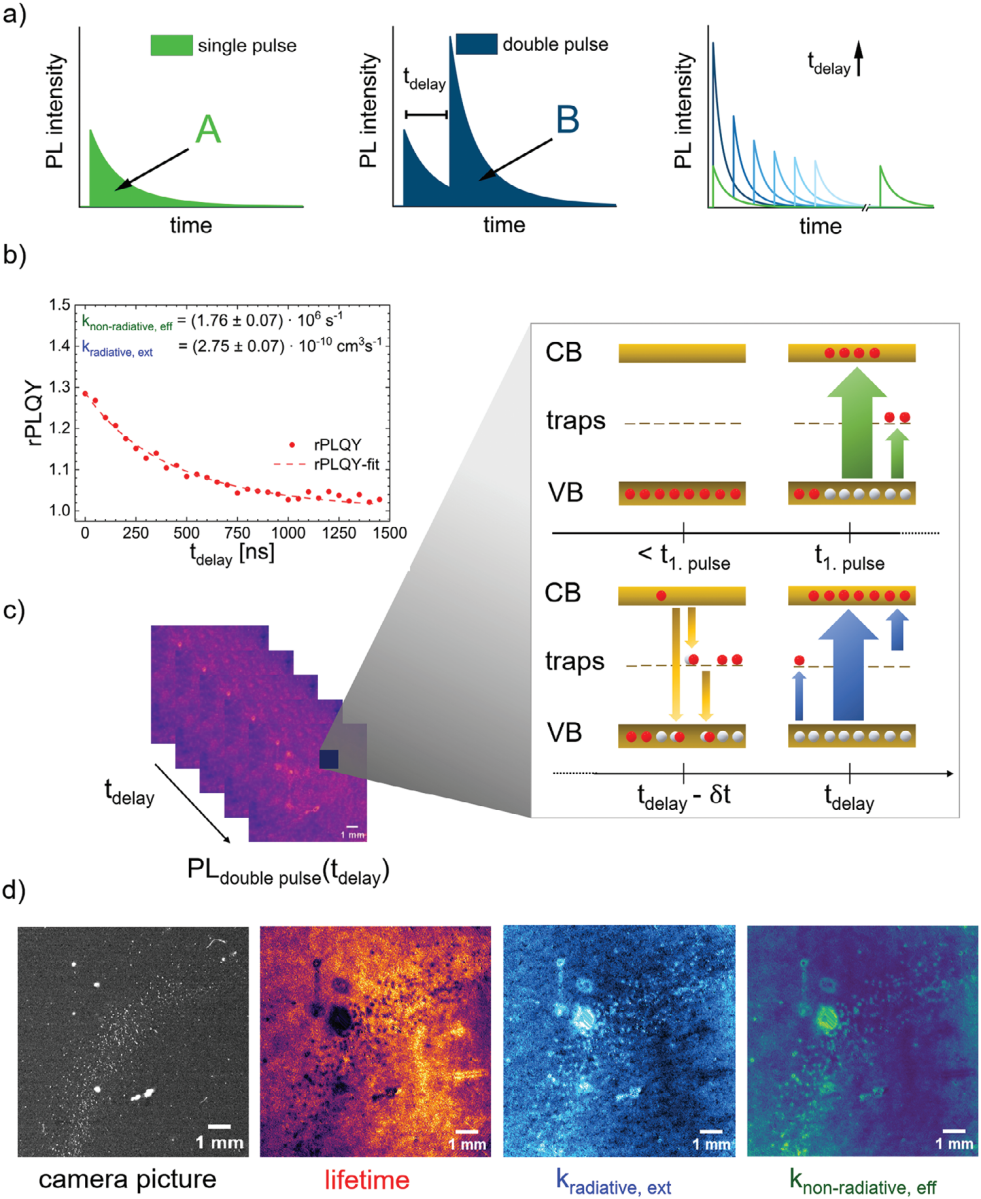
rPLQY‐imaging methodology. a) Schematic transient PL decay of a single (left) and double‐pulse (center) excited sample. Illustration of transient PL decay with varying delay times in different blue shades (right). b) Exemplary rPLQY versus delay time including fitting and resulting rate constants. c) PL‐images for varying delay times (left) and schematic illustration of charge carrier distribution before the first pulse (<*t*_1, pulse_), directly after the first excitation pulse (*t*_1, pulse_), before the second excitation pulse (*t*_delay_ *‐δt*) and directly after the second excitation pulse (*t*_delay_) (right). d) Exemplary raw camera image without excitation. Images, derived by the rPLQY method, show lifetime, external radiative, and effective non‐radiative rate constant after applying pixel‐wise fitting.

In the first step, we introduce the methodology of rPLQY‐imaging. The advanced image‐based characterization technique is tailored for thin film processing and facilitates an in‐depth understanding of charge carrier dynamics and absorber quality. This technique is based on a double‐pulse excitation routine with varying delay times between pulses. The rate constants are extracted after analysis using a model developed by Kaiser et al.,^[^ ^18^ ^]^ A schematic of the working principle is illustrated in **Figure** **2**a. Single pulse (SP) excitation initiates an exponential PL decay, which is immediately reset by a second pulse after a specified time delay (*t*_delay_) (see Figure 2a). With increasing *t*_delay_ of the second pulse, the electron density in the trap states decreases until all electrons have recombined into the valence band. For *t*_delay_ → ∞ the two decays will resemble those of two individual excitation pulses (see Figure 2a right, green traces). By systematically varying the delay time and observing the PL intensity from single and double‐pulse excitation, we can probe the free charge carrier dynamics initiated by the first pulse, at different excitation levels introduced by the second pulse (see Figure 2a right, blue traces). As shown in our previous work, the rPLQY can then be determined by the ratio of the time‐integrated PL intensity following double‐pulse excitation *B* to twice the time‐integrated PL intensity following single‐pulse excitation *A*.^[^ ^18^ ^]^

To intuitively understand the dependence of rPLQY on the rate constants, Figure 2c illustrates the excess charge carrier concentration in the states at each point in time during double‐pulse excitation. Prior to the first pulse, all electrons remain in the valence band. During the first pulse, absorption excites electrons to the conduction band, leaving holes in the valence band. After excitation, electrons start to recombine with the holes either radiatively or non‐radiatively via trap states.^[^ ^19^, ^20^ ^]^ As charge carriers continuously recombine, the excess charge carrier concentration changes dynamically. Therefore, the concentration at the moment of the second pulse depends on the delay time. Consequently, varying the delay time enables access to the charge carrier dynamics.

Based on the description above, the rPLQY depends on the effective non‐radiative and external radiative rate constants, *k*_non‐radiative, eff_ and *k*_radiative, ext_, the delay time *t*_delay_ and the initial density of the excitation pulse *N*_0, A_, and *N*_0, B_. The effective non‐radiative rate is the sum of Shockley‐Read‐Hall (SRH) recombination and, if applicable, surface recombination *k*_surf_.^[^ ^21^ ^]^ To minimize correlation in the fitting process, parameters of additional detailed mechanisms influencing SRH recombination rate, such as detrapping from shallow traps,^[^ ^9^ ^]^ are not explicitly determined in the model. However, these effects are implicitly included, as the model determines the overall effective non‐radiative rate. The external radiative recombination is the product of internal radiative recombination *k*_radiative, int_, and the sum of escape and parasitic absorption probability (*p*_e_ + *p*_a_).^[^ ^22^ ^]^ Given that *N*_0, A_, *N*_0, B_, and the delay time are experimentally accessible parameters, appropriate fitting allows for deriving the rate constants from a rPLQY data set, as detailed by Kaiser et al.^[^ ^18^ ^]^ In the following, we provide a brief overview of the main pillars of the model.

For the emission of free charge carriers, the PL intensity is directly proportional to the square of the charge carrier density *n*, expressed as PL = *k*_radiative, ext_ ∙ *n* ^2^.^[^ ^23^ ^]^ Introducing a second pulse after a given delay time *t*_delay_, the PL can be split into two parts: PL(*t* ≥ *t*_delay_) and PL(*t* < *t*_delay_). The PLQY for the single and double pulse scenario of a free carrier is the ratio of the total PL emitted and the initial density,^[^ ^24^, ^25^ ^]^ hence described as:

(1) $$\begin{array}{c} \mathrm{PLQ}Y_{\mathrm{SP}}=\frac{\int_{0}^{\infty }\mathrm{PL}\;\mathrm{dt}}{N_{0,A}}=A\\ \mathrm{PLQ}Y_{\mathrm{DP}}=\frac{\int_{0}^{\infty }\mathrm{PL}\;\mathrm{dt}}{N_{0,A}+N_{0,B}}=B \end{array}$$

Consequently, the rPLQY is expressed as:

(2) $$\mathrm{rPLQY}\;\left(t_{\mathrm{delay}}\right)=\frac{\mathrm{PLQ}Y_{\mathrm{DP}}\left(t_{\mathrm{delay}}\right)}{\mathrm{PLQ}Y_{\mathrm{SP}}\left(t_{\mathrm{delay}}\to \infty \right)}=\frac{B}{2\cdot A}$$

The complete mathematical expression is provided in Equation S1 (Supporting Infomation). Applying this model to the data leads to the rate constants of the sample investigated. Further insight into kinetic characteristics enables the lifetime τ expressed as τ = *k*_non‐radiative, eff_^−1^.^[^ ^21^ ^]^

Given the important role of charge carrier dynamics in evaluating perovskite film quality, we aim to transform this model into a spatially resolved imaging technique covering large areas. This method aims to enable precise characterization and troubleshooting of the perovskite layer during its fabrication process. To meet laboratory requirements for an effective characterization imaging method, our setup focuses on ease of implementation, rapid data acquisition, and adaptability for large‐scale sample areas. Detailed descriptions of the setup and analysis procedure are available in the methods section below. Achieving a fully spatially resolved charge carrier dynamics characterization of thin films (see Figure 2d) requiring only a delay generator, two pulsed light sources, and a camera emphasizes the scientific applicability of this method. Avoiding spectral resolution eliminates the need for a spectrometer, highlighting its ease of implementation while simultaneously reducing space and costs. Moreover, adjusting the camera zoom allows flexibility in the investigated area, while data acquisition and analysis times depend on acquisition parameters and region of interest (ROI) size. With further software optimization and tailored system configurations, rate‐constant images could be generated within minutes.

### 2.2 Validation

#### 2.2.1 Experimental Validation of Perovskite Thin Film Rate Constants

We show the method's reliability by comparing its results against the established TCSPC technique. This comparison highlights the capability of our technique to capture the charge carrier dynamics of perovskite thin films.

**Figure 3:**
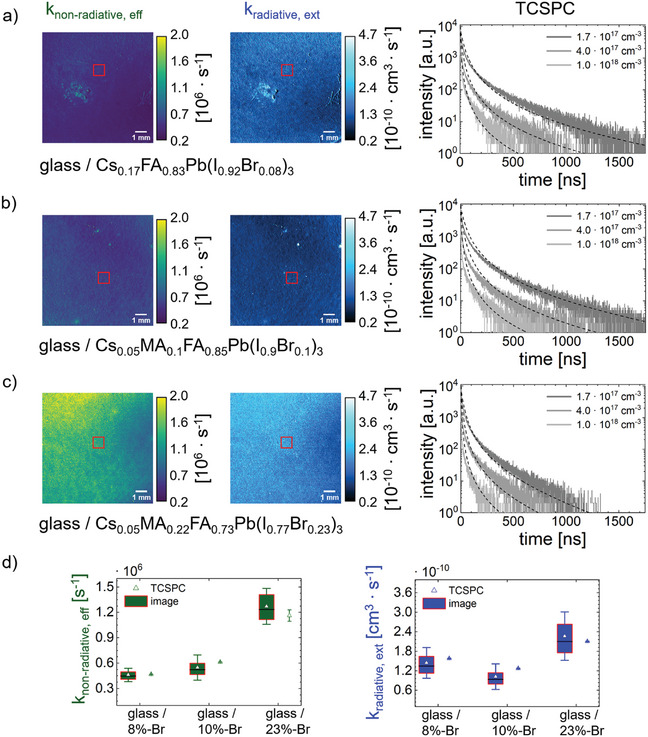
Experimental validation of perovskite thin film rate constants. a–c) Images of external radiative and effective non‐radiative recombination rate obtained by rPLQY‐imaging of glass/perovskite stacks and PL decays at different initial densities obtained by time‐correlated single photon counting (TCSPC) measurements. a) Cs_0.17_FA_0.83_Pb(I_0.92_Br_0.08_)_3_, labeled as 8%‐Br b) Cs_0.05_MA_0.1_FA_0.85_Pb(I_0.9_Br_0.1_)_3_, labeled as 10%‐Br c) Cs_0.05_MA_0.22_FA_0.73_Pb(I_0.77_Br_0.23_)_3_, labeled as 23%‐Br d) Comparison between rPLQY‐imaging and TCSPC results of each stack.

Three different perovskite thin film compositions Cs_0.17_FA_0.83_Pb(I_0.92_Br_0.08_)_3_ (8%‐Br), Cs_0.05_MA_0.1_FA_0.85_Pb(I_0.9_Br_0.1_)_3_ (10%‐Br) and Cs_0.05_MA_0.22_FA_0.73_Pb(I_0.77_Br_0.23_)_3_ (23%‐Br) with a thickness of (400–500) nm are deposited directly on the glass to isolate their pure kinetic characteristics without interference from additional functional layers such as electrodes or charge transport layers (CTLs). TCSPC measurements are conducted using a 532 nm pulsed laser at varying intensities, leading to initial densities *N*_0_ of 1.7 × 10^17^ cm^−3^, 4.0 × 10^17^ cm^−3^, and 1.0 × 10^18^ cm^−3^ around the sample center, as indicated by the red ROI (see **Figure** **3**a–c). Using three different initial densities is essential for performing global fitting (see Equation S2, Supporting Infomation) to obtain accurate recombination rates.^[^ ^9^, ^26^ ^]^ Given the disparity between laser spot and sample size, limiting comparisons to the red ROI ensures accuracy (see Figure 3a–c). Comparing results obtained by rPLQY‐imaging and the benchmark method shows a congruent trend within the acceptable margin of error, confirming its validity (see Figure 3d).

Next, we present a detailed comparison of the rate constants obtained from rPLQY‐imaging and TCSPC measurements, revealing deviations ∆*k*_i, j_ (i ∈ {non‐radiative eff, radiative ext} and j ∈ {8%‐Br, 10%‐Br, 23%‐Br}) in the effective non‐radiative and external radiative recombination rates across the investigated perovskite thin films. The 8%‐Br and 23%‐Br perovskite show minimal deviations for both rate constants below 10% with: ∆*k*_non‐radiative eff, 8%‐Br_ = 1% and ∆*k*_radiative ext, 8%‐Br_ = 8%, and ∆*k*_non‐radiative eff, 23%‐Br_ = 9% and ∆*k*_radiative ext, 23%‐Br_ = 7%, respectively, as shown in Figure 3d. Meanwhile, the 10%‐Br perovskite shows the highest deviations in effective non‐radiative (∆k_non‐radiative eff, 10%‐Br_ = 10%) and external radiative recombination (∆*k*_radiative ext, 10%‐Br_ = 20%). Rate constant errors up to 20% are common in literature, indicating acceptable measurement success.^[^ ^27^, ^28^ ^]^

Previous research by Ruiz–Preciado et al.,^[^ ^29^ ^]^ compared the performance of the 10%‐Br and 23%‐Br perovskite, favoring 10%‐Br due to its ability to achieve a perovskite / CuInSe_2_ (CIS) tandem device PCE approaching 25%. Even though the 23%‐Br perovskite exhibits an over 50% higher radiative recombination rate than 10%‐Br (see Figure 3d), the superior performance of the 10%‐Br perovskite can be attributed to its lower non‐radiative recombination rate.^[^ ^30^ ^]^ These findings show the importance of quantitatively determining rate constants for drawing direct and accurate conclusions regarding sample quality. In addition, the rPLQY‐imaging technique provides a notable advantage over the single‐spot TCSPC method, as illustrated in the case of the 23%‐Br perovskite, which exhibits the highest variance of the rPLQY‐imaging results. We attribute this aspect to an inhomogeneous thin film deposition (see Figure 3c,d). Imaging helps detect inhomogeneities in the absorber film, which are of critical importance in the processing of large‐area devices. TCSPC measurements at various spots across the sample cannot achieve our setup's 7.9 µm spatial resolution precision, given that the TCSPC laser spot size is 1 mm^2^. Even TCSPC systems capable of achieving this resolution fail to deliver results in a reasonable timeframe, particularly considering the variability in initial densities required for global fitting. Given the potential presence of defects in the thin film sample, selecting statistically relevant areas becomes essential to maintain the accuracy of mean results. This circumstance is addressed by our rPLQY‐imaging method's ability to reveal such imperfections.

In summary, this section highlights the validity and accuracy of rPLQY‐imaging, as it provides results consistent with those obtained by TCSPC measurements. This agreement underscores the suitability of the rPLQY‐imaging method for scientific applications. Ultimately, these findings highlight the significance of proper experimental techniques and data analysis in advancing our understanding of perovskite materials for various applications.

#### 2.2.2 Validation of Rate Constants in Complex Sample Configurations via Simulation Study

In this section, we emphasize the ability of our imaging characterization method to derive charge carrier dynamics from various half‐stack configurations – an essential aspect for industrial and scientific applications. We demonstrate this by conducting numerical experiments, showcasing the method's effectiveness in extracting these crucial optoelectronic thin film characteristics.

We opted for a numerical experiment over traditional experimentation due to its inherent capability to efficiently explore a wider array of scenarios. Unlike conventional experiments, which are time‐consuming and constrained by resources, numerical simulations offer the flexibility to model various conditions comprehensively. Given the diverse effects of different perovskite compositions, electrodes, and CTLs on kinetic properties,^[^ ^21^ ^]^ we aim to establish a range of possible *k*_non‐radiative, eff_ and *k*_radiative, ext_ combinations. Demonstrating the functionality of our model within this range implies its suitability for various half‐stack configurations.

Our simulation study starts with stage 1, focusing on defining input parameters that accurately reflect the behavior of the observed sample, followed by raw data simulation. Stage 2 involves applying the model to the raw data, and stage 3 compares the output to the input. If the deviation between those two is minimal, the simulation study counts as a success.^[^ ^31^ ^]^ To determine appropriate input parameters for stage 1, we first need to understand how the kinetics of the perovskite are affected by the addition of electrodes and CTLs. Typical ranges for *k*_non‐radiative, eff_ are between 10^5^ and 10^9^ s^−1^ for bulk perovskites, while *k*_radiative, ext_ typically ranges between 10^−12^ and 10^−7^ cm^3^ s^−1^.^[^ ^32^ ^]^ The presence of electrodes and CTLs introduces complexities, significantly influencing recombination dynamics, depending on the interface recombination velocity *S*, perovskite thickness *d*, and initial charge carrier density *N*_0_.^[^ ^21^, ^33^, ^34^ ^]^ After excitation, charge carriers accumulate within the CTL and recombine either at the perovskite/CTL interface, which is more likely for high *S* or in the perovskite bulk, which is more likely for low *S*. The interfacial recombination velocity ranges between 10^−2^ cm and 10^3^ cm s^−1^, with *S* = (10^−1^–10^2^) cm s^−1^ being typical reported values.^[^ ^21^, ^33^, ^35^, ^36^ ^]^ The input of the non‐radiative rate constant can be described as:

(3) $$k_{\mathrm{non}-\mathrm{radiative},\;\mathrm{eff},\;\mathrm{input}}=k_{\mathrm{non}-\mathrm{radiative},\;\mathrm{bulk}}+\frac{S}{2\cdot d}$$

where *k*_non‐radiative, bulk_ represents the bulk non‐radiative rate constant, which we set to 1.7 × 10^7^ s^−1^ and *d* to 400 nm as it represents typical perovskite thin film thicknesses.^[^ ^34^, ^37^ ^]^

**Figure 4:**
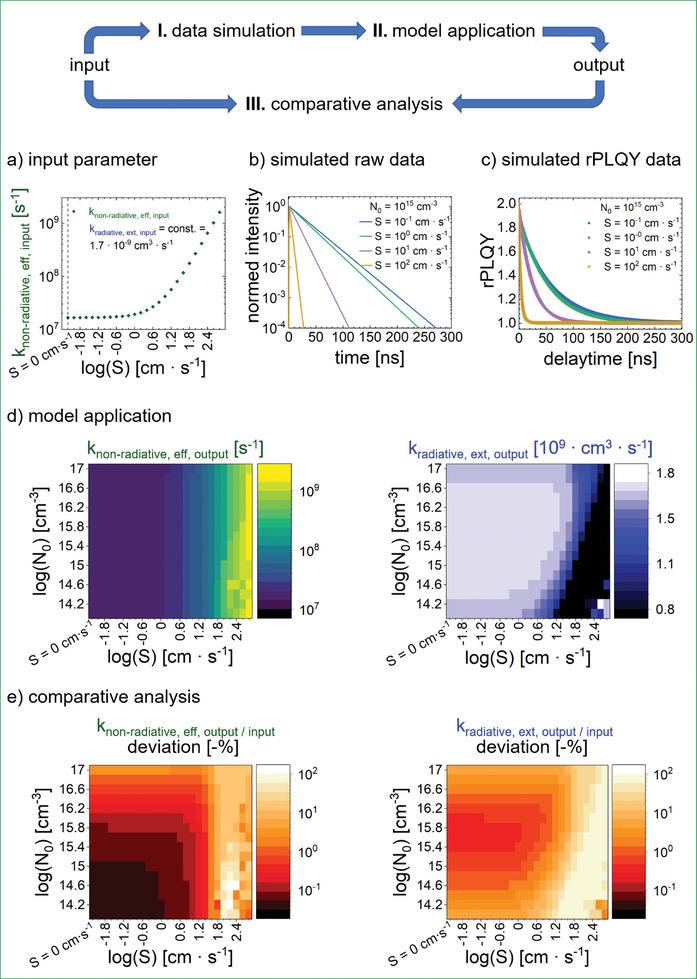
Validation via a simulation study. a) Input parameters of external radiative and effective non‐radiative rate constants *k*_radiative, ext_ and *k*_non‐radiative, eff_ in dependence on surface velocity *S* for simulation. b) Simulated double‐pulse time decays at constant initial density **N**_0_ and varying input parameter *S*. c) rPLQY curves at constant initial density *N*_0_ and varying input parameter S. d) Output parameters of external radiative and effective non‐radiative rate constants with varying initial densities *N*_0_ and input parameter *S*. e) Comparative analysis of output to input parameters *k*_non‐radiative, eff_ and *k*_radiative, ext_ in dependence of *N*_0_ and *S*.

By systematically varying *S*, we establish input conditions that encompass a wide range of parameter combinations, thereby facilitating assessments of half‐stack applicability. The simulated input parameters cover a wide range, with *k*_non‐radiative, eff, input_ spanning from 1.7 × 10^7^ to 1.6 × 10^9^ s^−1^, containing 26 discrete data points. The *k*_radiative, ext, input_ is set to 1.7 × 10^−9^ cm^3^ s^−1^ (see **Figure** **4**a) and the initial densities of the lasers log(*N*_0_) ranges from 14.0 to 17.0 in 0.2 increments, yielding more than 400 distinct scenarios. The delay time varies from 0 ns to 500 ns, with a resolution of 0.01 ns. Subsequently, decay curves are simulated based on these parameters following Equation S2 (Supporting Infomation). An exemplary decay curve for *N*_0_ = 10^15^ cm^−3^, varying *S* and a second pulse delay time of 1 ns, is shown in Figure 4b. Consistent with theoretical expectations, an increased effective non‐radiative recombination rate leads to a faster decay process.^[^ ^21^ ^]^ After the simulation of the raw data set, the processing is applied according to Equation (2), resulting in rPLQY curves as shown in Figure 4c, thereby concluding stage 1.

In stage 2, the data is analyzed using model Equation S1 (Supporting Infomation), producing *k*_non‐radiative, eff, output_, and *k*_radiative, ext, output_ for each scenario, as illustrated in Figure 4d. A visual comparison between Figure 4a,d immediately demonstrates the accurate trend in the results*. k*_non‐radiative, eff, output_ exhibits a trend of increase with rising *S*, while remaining unaffected by the initial charge carrier density, as evident from a consistent color transition from dark green to yellow along the vertical axis. In contrast, given the constant *k*_radiative, ext, input_, the output color map should demonstrate uniformity across the applied range, which holds true for *S* values ranging from (0–10^1.2^) cm^3^ s^−1^ and almost all initial densities spanning from 10^14^ to 10^17^ cm^−3^ (see Figure 4d).

To unambiguously interpret the results, we proceed to stage 3 by comparing the input to the output (see Figure 4e). It is important to note the logarithmic scale of the color bar, where darker red and orange regions indicate deviations of less than 1% and 10%, respectively. Notably, regions exhibiting deviations >50% are illustrated as light yellow, observed specifically for *S* ≥10^2^ cm^3^ s^−1^ across all *N*_0_ values and *S* ≥10^1.2^ cm^3^ s^−1^, *N*_0_< 10^15^ cm^−3^ for *k*_radiative, ext_. While these areas represent extreme cases that push the limits of our model, they are still valuable for understanding potential outliers. Typically, decay experiments are conducted with initial densities ranging from 10^15^ to 10^16^ cm^−3^,^[^ ^38^, ^39^ ^]^ so these extreme cases are not expected to form a significant problem. However, it is crucial to consider them in our analysis.

The numerical experiment demonstrates the method's suitability with half‐stack configurations, evident from the minimal deviation between the input and output parameters. These findings underscore its potential for industrial and scientific applications.

### 2.3 Example of Applications for the rPLQY‐Imaging Method

#### 2.3.1 Improved Quality Analysis of Thermally Treated and Hot‐Pressed Layers on a Single Substrate Compared to Established PL‐Imaging Techniques

In this chapter, we emphasize two essential attributes of an imaging characterization technique: i) the capacity to distinguish between selected areas, and ii) the enhanced information depth compared to other PL techniques, enabling the estimation of variations in escape and parasitic absorption probabilities. The following study explores these features to conduct a detailed surface analysis and spatial differentiation.

**Figure 5:**
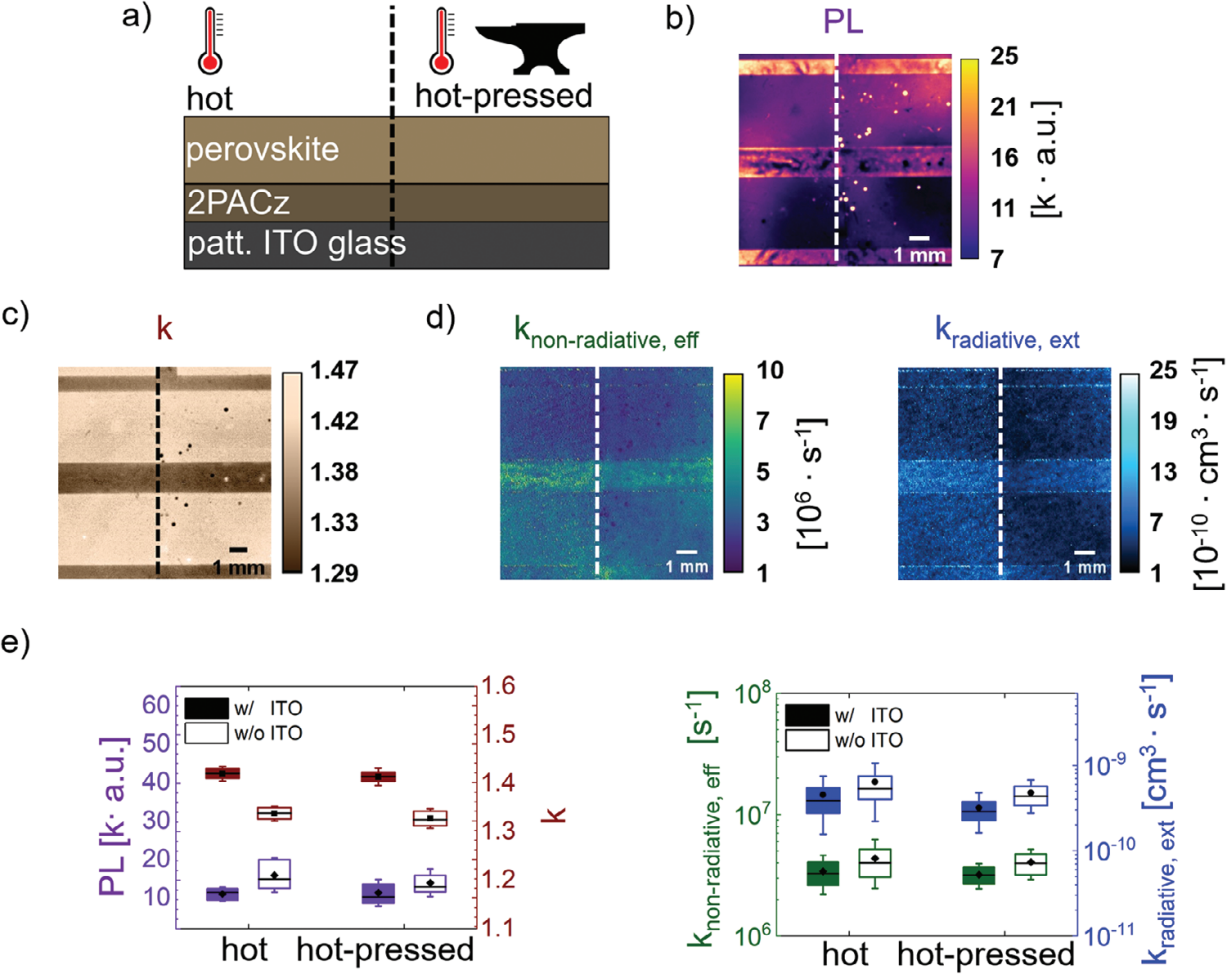
Comparison of PL‐based imaging techniques. a) Illustration of the sample configuration and treatment. The left side of the sample is treated with heat (hot) and the right side with heat and pressure (hot‐pressed) b) PL image, c) *k*‐image, d) effective non‐radiative and external radiative rate constant image obtained by rPLQY‐imaging acquired from absorber side of the same sample. e) Average values of the characteristics of each area obtained by PL‐imaging, *k*‐imaging, and rPLQY‐imaging, respectively.

We employ three distinct imaging techniques—PL‐imaging, *k*‐imaging, and rPLQY‐imaging—to analyze the same sample and compare their respective information depth (see Figure 1). The sample comprises glass / patterned ITO / 2PACz / Cs_0.1_(MA_0.17_FA_0.83_)_0.9_Pb(I_0.83_Br_0.17_)_3_. To illustrate surface quality disparities across four designated areas (see **Figure** **5**a), half of the sample is hot‐pressed at 90 °C and 80 MPa for 5 min, while the other half is only subject to the high temperature, as reference. The areas with and without ITO contain information of ≈380 000 and 168 000 data points, respectively (see Figure S1, Supporting Infomation). The hot‐pressing process is applied to improve the morphology of perovskite absorber film by increasing its grain size and decreasing its surface roughness, as described in our previous work by Roger et al.^[^ ^40^ ^]^

During sample optimization, PL‐based imaging techniques are crucial for understanding spatially resolved charge carrier recombination. However, information depth varies among methods. Optical artifacts like stray light, reflections, and inhomogeneous illumination can influence traditional PL imaging. As a higher PL signal of a sample is commonly associated with higher layer quality,^[^ ^9^ ^]^ these effects complicate result interpretation. Figure 5b illustrates this difficulty, as the sample shows inhomogeneities, suggesting differences between the reference and hot‐pressed sides of the sample. We attribute the vertical line in the middle to a reflection artifact. Furthermore, the hot‐pressed side exhibits spots with enhanced PL signal, signifying defects that potentially emerged due to the hot‐pressing step. However, comparing the PL values of each area (see **Table** **1**) indicates no significant quality differences between both sides.

**Table 1:** PL‐imaging, *k*‐imaging, and rPLQY‐imaging result distribution of four distinct areas of a half‐stack configured sample treated half with heat (hot) and half with heat and pressure (hot‐pressed) (see Figure 5).

	Hot		Hot‐Pressed	
	w/ ITO	w/o ITO	w/ ITO	w/o ITO
PL [k ∙ a.u.]	11.0 ± 1.8	15.9 ± 4.5	11.3 ± 3.5	13.9 ± 3.6
*k*	1.42 ± 0.02	1.34 ± 0.02	1.41 ± 0.02	1.33 ± 0.02
*k*_non‐radiative, eff_ [10^6^ ∙ s^−1^]	3.4 ± 1.2	4.4 ± 1.9	3.2 ± 0.7	4.1 ± 1.1
*k*_radiative, ext_ [10^−10^ ∙ cm^3^ ∙ s^−1^]	4.5 ± 2.9	6.3 ± 4.2	3.2 ± 1.6	4.7 ± 2.0

Intensity‐dependent PL measurements reveal further information on recombination kinetics, with PL following PL∝*I^k^*, where *k* ranges from 1 to 2, indicating the dominant recombination path.^[^ ^15^, ^16^, ^17^ ^]^ *k*‐imaging is robust against optical artifacts and offers quantitative results, enhancing information depth compared to conventional PL‐imaging.^[^ ^11^ ^]^ However, interpreting the *k*‐value remains challenging, as no discernible differences can be visually recognized between the hot and hot‐pressed sides (Figure 5c). The hot‐pressed and heat‐treated sides show similar overall *k*‐values (see Table 1) for both areas with and without ITO. Complexity arises from the fact that the *k*‐value serves as an indicator of quality, but does not represent the ratio of the rate constants.

The highest information depth is ensured by rPLQY‐imaging, offering direct quantification of both rate constants. Figure 5d,e exhibit noticeable differences between the hot and hot‐pressed sides, showing a decrease in *k*_radiative, ext_ for the areas with and without ITO on the hot‐pressed side by 29% and 25%, respectively, while *k*_non‐radiative, eff_ remains relatively constant. Moreover, the relative standard deviation decreases for the hot‐pressed side by 13%–25% (see Table 1), as evidenced by a smoother distribution in the images. This reduction in deviation is attributed to reduced scattering effects resulting from an increased grain size and lower surface roughness on the hot‐pressed side (see Figure S2, Supporting Infomation). Considering that a lower non‐radiative rate promises better quality, it seems contradictory that it stays constant on both sides. The combination of imaging different areas and acquiring the respective rate constants enables a more detailed analysis to resolve this contradiction. Given that the only difference between the sides is the pressing step, specifically leading to increased grain size and reduced surface roughness for the hot‐pressed area, this suggests that the differences are superficial with no significant impact on the intrinsic perovskite bulk properties.^[^ ^41^, ^42^ ^]^ The experimentally obtained external radiative recombination is a product of the internal radiative recombination rate *k*_radiative, int_ and the sum of escape and parasitic absorption probabilities, *k*_radiative, ext_ = (*p*_e_ + *p*_a_) ⋅ *k*_radiative, int_.^[^ ^9^, ^43^ ^]^ Assuming consistent material properties, *k_radiative, int_* remains constant. Thus, the observed change in *k*_radiative, ext_ must be a result of a variation in the sum (*p*_e_ + *p*_a_). Without ITO, *k*_radiative, ext_ = *p*_e_ ⋅ *k*_radiative, int_, (with *p*_a_ = 0), because there is no parasitically absorbing electrode.^[^ ^44^, ^45^, ^46^ ^]^ Analysing the relative escape probabilities *k*_radiative, ext, w/o ITO, hot_ / *k*_radiative, ext, w/o ITO, hot‐pressed_ = *p*_e, w/o ITO, hot_ / *p*_e, w/o ITO, hot‐pressed_ = 1.34 (see Table 1) implies that the escape probability decreased by 25.4% relatively on the hot‐pressed side. This reduction in escape probability is attributed to lower surface roughness, leading to reduced light scattering at the film surface and consequently lower photon outcoupling, as discussed in our previous work by Fassl et al.^[^ ^14^ ^]^ Repeating these calculations for the area with ITO shows a reduction in *k*_radiative, ext, w/ ITO, hot‐pressed_ = 0.711 ⋅ *k*_radiative, ext, w/ ITO, hot_ by 28.9% on the hot‐pressed side (see Table 1). This slightly enhanced overall reduction in (*p*_e, w/ ITO, hot‐pressed_ + *p*_a, w/ ITO, hot‐pressed_) is attributed to additional parasitic absorption in the presence of the ITO layer. Thus, the presented rPLQY‐imaging technique can be used to identify relative differences in the escape and parasitic absorption probabilities of a sample.

This study underscores the superiority of the rPLQY‐imaging technique over other PL‐based methodologies. By directly providing results of the parameters of interest, rPLQY‐imaging ensures high interpretation depth, making it beneficial for various applications in industry and science.

#### 2.3.2 Estimation of Surface Recombination Velocity at Perovskite/CTL Interface

We demonstrate the capabilities of the rPLQY‐imaging technique when applied to strategically designed sample configurations. Investigating a sample partially deposited with C_60_ allows for an estimation of the surface recombination velocity at the perovskite / CTL interface. Accessing this parameter emphasizes the method's potential for sample optimization.

Non‐radiative surface recombination is a common loss mechanism in perovskite solar cells.^[^ ^21^, ^47^, ^48^, ^49^ ^]^ This recombination occurs primarily due to defects at the interface between the perovskite absorber and the CTL,^[^ ^50^ ^]^ as well as due to energy level offsets between these layers.^[^ ^51^ ^]^ In the case of an electron transport layer (ETL) like C_60_, this offset allows charges from the perovskite's valence band to recombine with those in the lowest unoccupied molecular orbital level of the ETL.^[^ ^51^ ^]^ There have been several attempts to reduce surface recombination by introducing passivation layers^[^ ^50^, ^52^ ^]^ or aligning the energy levels of perovskite and CTLs.^[^ ^51^, ^53^ ^]^ Quantitively, surface recombination can be described by its rate

(4) $$k_{\mathrm{non}-\mathrm{radiative},\;\mathrm{surf}}=\frac{S}{2\cdot d}$$

where *S* is the surface recombination velocity and *d* is the perovskite film thickness.^[^ ^21^ ^]^ Krogmeier et al.,^[^ ^33^ ^]^ developed an accurate approach for determining the surface recombination velocity by performing transient PL measurements across a range of fluences and fitting the resulting data to their model. The extracted parameters from the global fit are validated through comparison with simulations conducted within a specific parameter space around the fit results. The final parameter set is selected based on minimizing the overall error across all fluence levels.

**Figure 6:**
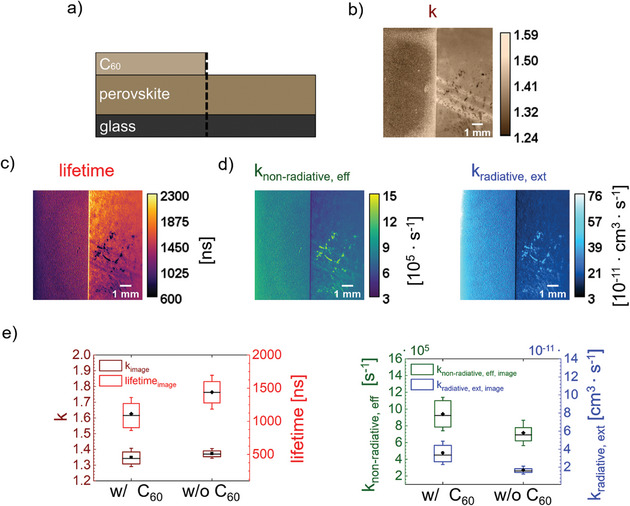
Estimation of surface recombination velocity. a) Schematic illustration of sample configuration. C_60_ is additionally deposited on the left‐hand side of the sample b) *k*‐image of the sample c) Lifetime image of the sample determined by the inverse of effective non‐radiative recombination rate. d) Effective non‐radiative and external radiative rate constant image of sample obtained by rPLQY‐imaging. e) Average values of each area and parameter obtained, k‐imaging and rPLQY‐imaging, respectively.

The presented rPLQY‐imaging method provides a valuable approach for estimating the surface recombination velocity by determining the effective non‐radiative recombination rate, which includes bulk and surface contributions (see Equation 3). By leveraging the spatial resolution of the imaging technique, examining a sample comprised of glass / (Cs_0.22_FA_0.78_Pb(I_0.85_Br_0.15_)_3_ + 5% MAPbCl_3_) with C_60_ deposited on half of its surface (see **Figure** **6**a), enables investigation of surface recombination dynamics. Using Equations (3) and (4), the difference of *k*_non‐radiative, eff_ of both sides leads to *k*_non‐radiative, surf_, assuming that *k*_non‐radiative, bulk_ is constant throughout the sample. With known perovskite thickness *d*, the surface recombination velocity *S* can be approximated.

*k*‐imaging of the sample reveals comparable *k*‐values for both sides (see Figure 6b,e, and **Table** **2**), with only a 1.4% reduction and twice the standard deviation on the C_60_ side. This analysis suggests that both sides demonstrate similar dominant recombination pathways. We note that based on the sample configuration, a more significant decrease in the *k*‐value due to surface recombination was expected. As previously discussed, interpretation is challenging since multiple recombination processes can contribute to a single *k*‐value.

**Table 2:** *k*‐imaging and rPLQY‐imaging result distribution of two distinct areas of a glass/perovskite sample with additional C_60_ deposited half on the sample (see Figure 6).

	w/ C_60_	w/o C_60_
*k*	1.35 ± 0.06	1.37 ± 0.03
lifetime [ns]	1110 ± 249	1438 ± 256
*k*_non‐radiative, eff_ [10^5^ ∙ s^−1^]	9.4 ± 2.0	7.2 ± 1.5
*k*_radiative, ext_ [10^−11^ ∙ cm^3^ ∙ s^−1^]	3.6 ± 1.3	1.7 ± 0.4

The rPLQY‐imaging method enhances measurement accuracy by providing quantitative values for the rate constants. The C_60_ side exhibits an increased *k*_radiative, ext_ by a factor of 2.1 (see Figure 6d,e, and Table 2). Considering that the layer stack is the same on both sides up to the C_60_ layer, we assume that *k*_radiative, int_ remains constant. Consequently, the observed increase in *k*_radiative, ext_ is attributed to variations in the combined probabilities (*p*_e_ + *p*_a_). An increase in *k*_radiative, ext_ by such factors is also observed by Krogmeier et al.^[^ ^33^ ^]^ The escape probability *p*_e_ can be described by the geometry of the escape cone, which defines the region from which photons can leave the absorber at the interface.^[^ ^54^ ^]^ This geometry is dependent on the refractive indices *n*_i_ of the perovskite layer and the overlying material.^[^ ^55^ ^]^ Since C_60_ has a refractive index of 2.2, more than twice as *n*_air_, the resulting escape cone is enlarged. Moreover, parasitic absorption *p*_a_ is increased for the C_60_ side, considering its non‐negligible absorption in the visible wavelength region together with waveguiding effects.^[^ ^14^, ^56^, ^57^ ^]^

The effective non‐radiative rate *k*_non‐radiative, eff_ demonstrates an increase of 28% on the C_60_ side (see Figure 6d,e, and Table 2). Quantifying the difference between the mean values of both sides and applying Equation (4), leads to an estimate of *S*≈18 cm s^−1^. As the calculation does not consider possible variations in *k*_non‐radiative, bulk_, and inhomogeneities within the sample, the resulting surface velocity serves as a useful approximation for subsequent investigations. The decrease in lifetime (see Figure 6c,e, and Table 2) on the C_60_ side is attributed to increased surface recombination.^[^ ^47^ ^]^

The principal advantage of the presented rPLQY‐imaging method is its capability to quantitatively assess essential characteristics of charge carrier dynamics. Furthermore, when applied to strategically designed experiments and samples, this technique can provide additional insights, such as estimating the surface recombination velocity. This capability facilitates not only the optimization of the absorber material but also of the interface characteristics.

## 3 Conclusion

This work introduces a non‐invasive and scalable PL‐based imaging technique for assessing charge carrier dynamics in perovskite thin films. The technique is based on time‐integrated PL‐imaging and double‐pulse excitation with varying pulse‐to‐pulse delay. rPLQY images obtained from a series of double‐pulse excitation experiments yield essential optoelectronic characteristics of perovskite absorbers, such as external radiative and effective non‐radiative recombination rate constants and corresponding lifetimes, all spatially resolved. We demonstrate the capability of the rPLQY‐imaging method to deliver consistent results for perovskite thin films on glass with those obtained by TCSPC measurements. A simulation study further validates the method's suitability for complex sample configurations containing additional functional layers, such as electrodes and charge transport layers, which can affect the perovskite's optoelectronic characteristics. The method's spatial resolution provides insights into inhomogeneities and defects within the samples. By directly quantifying charge carrier dynamics, the method allows for a detailed characterization of perovskite materials.

Additionally, a comparison of the rPLQY‐method with traditional PL‐imaging and *k*‐imaging is conducted on a half‐stack sample including patterned ITO which is hot‐pressed on one half, while the other half is only subject to heat. Despite the significant recrystallization of the film surface under hot‐pressing, traditional PL‐imaging and *k*‐imaging techniques fail to detect differences. In contrast, the rPLQY‐imaging technique effectively demonstrates a smoother parameter distribution on the hot‐pressed side. Furthermore, it reveals changes in recombination rates across all areas, leading to the identification of relative differences in escape and parasitic absorption probabilities.

Finally, we show that applying this method to samples partially deposited with a charge transport layer allows for the estimation of surface recombination velocity. Leveraging the spatially resolved effective non‐radiative recombination rate and assuming consistent non‐radiative bulk contributions throughout the sample, enables the assessment of surface characteristics, providing valuable insights for optimizing interface quality.

Whether used in scientific or future industrial contexts, an easy, non‐invasive implementation and high throughput are crucial requirements for a characterization method. The presented rPLQY‐imaging technique meets these requirements, as it requires only two pulsed lasers, a delay generator, and a camera, making it space‐efficient and cost‐effective. In conclusion, the presented rPLQY‐imaging technique is a valuable tool for scientific applications, offering significant advantages in the characterization and optimization of large‐area perovskite thin films.

## 4 Experimental Section

### Materials

2PACz (TCI, CAS: 20999‐38‐6), Lead iodide (PbI_2_, TCI, CAS: 10101‐63‐0), Lead bromide (PbBr_2_, TCI, CAS: 10031‐22‐8), Formamidinium iodide (FAI, Dyenamo, CAS: 879643‐71‐7), Methylamonium bromide (MABr, Greatcell Solar, CAS: 6876‐37‐5), Cesium Iodide (CsI, ABCR, CAS: 7789‐17‐5), Methylammonium chloride (MACl, CAS: 593‐51‐1, Lumtec), Lead chloride (PbCl_2_, CAS: 7758‐95‐4, TCI), Fullerene‐C_60_ (C_60_, Sigma–Aldrich, CAS: 99685‐96‐8), Bathocuproine (BCP, Lumescence Technology, CAS: 4733‐39‐5). Solvents including N,N‐dimethylformamide ≥99.9% (DMF, CAS: 68‐12‐2), Dimethyl sulfoxide anhydrous ≥99.9% (DMSO, CAS: 67‐68‐5), ethyl acetate (EA, 141‐78‐6) and Chloro Benzene anhydrous 99.8% (CB, CAS: 108‐90‐7) were ordered from Sigma–Aldrich. Ethanol absolute 99.8% was ordered from VWR Chemicals.

### Sample Preparation—Substrates and CTL

(Un‐)patterned glass and ITO substrates (sheet resistance 15 Ω sq^−1^, Luminescence Technology, CAS: 50926‐11‐9) were cut in 16 mm × 16 mm and cleaned by ultra‐sonication using deionized water, acetone, and isopropanol in an ultrasonic bath for 10–15 min each. Afterward, the substrates were activated with oxygen plasma for 3–5 min before deposition. For the HTL, a 1.63 mmol/l 2PACz solution in ethanol was spin‐coated at 3000 rpm for 30 sec and annealed at 100 °C for 10 min in N_2_ atmosphere. For the ETL 20 nm of C_60_ is evaporated using a half‐half mask, covering half of the active area with C_60_.

### Perovskite Fabrication—Cs_0.17_FA_0.83_Pb(I_0.92_Br_0.08_)_3_ (see Figure 3)

The perovskite solution was prepared by dissolving PbI_2_ (444 mg), PbBr_2_ (46 mg), CsI (46 mg), and FAI (143 mg) in 1 mL DMF:DMSO (4:1 volume ratio). Then it was spin‐coated at 1000 rpm (2000 rpms^−1^) for 10 s and 5000 rpm (2000 rpms^−1^) for 40 s. 20 s after the start of the second spin‐coating step, the substrate was washed with 150 µl chlorobenzene. The perovskite film was then annealed at 100 °C for 30 min in an inert atmosphere.

### Perovskite Fabrication—Cs_0.05_MA_0.1_FA_0.85_Pb(I_0.9_Br_0.1_)_3_ (see Figure 3)

The perovskite precursor solution was prepared by first dissolving PbI_2_ (1.3 m, 602.5 mg) in a mixture of DMF:DMSO (4:1 v/v, 800µl:200µl). The PbI_2_ solution was kept on a hotplate at 130 °C for 15 min and added to a mixture of PbBr_2_ (0.14 m, 51,75 mg), MABr (0.14 m, 15,8 mg) and FAI (1.2 m, 206 mg). The solution was then stirred until fully dissolved. From a CsI (1.5 m, 390 mg) in DMSO (1 mL) solution, the Csl (0.07 m, 46,7 µL) is added to the solution. To spincoat the perovskite films 100 µl of the precursor solution was dispensed on the substrates, which were then spun at 1000 rpm (500 rpm s^−1^) for 10 s and 5000 rpm (2000 rpm s^−1^) for 20 s. 25 s after the start, CB (150 µL) was dispensed onto the center of the spinning substrate. The samples were then annealed at 140 °C for 10 min in a nitrogen atmosphere.

### Perovskite Fabrication—Cs_0.05_FA_0.72_MA_0.22_Pb(I_0.77_Br_0.23_)_3_ (see Figure 3)

1.53 M Cs_0.05_FA_0.72_MA_0.22_Pb(I_0.77_Br_0.23_)_3_ perovskite precursor solution was prepared by dissolving a mixture of PbI_2_, PbBr_2_, Csl, MABr, and FAI in the solvents of DMF:DMSO (volume‐ratio being 4:1). The perovskite layers were deposited by spin coating in a nitrogen glovebox at 1000 rpm (200 rpm^−1^s^−1^) for 10 s and 5000 rpm (2000 rpm^−1^s^−1^) for 30 s, as well as 150 µl ethyl acetate as antisolvent was dropped 13 s before the end of the second step. Afterward, the perovskite layers were annealed at 100 °C for 30 min.

### Perovskite Fabrication—Cs_0.1_(MA_0.17_FA_0.83_)_0.9_Pb(I_0.83_Br_0.17_)_3_ (see Figure 5)

Two solutions were prepared. First, CsI (1.5 mol) in DMSO. Second, FAI (1 mol), PbI_2_ (1.1 mol), MABr (0.2 mol), and PbBr_2_ (0.22 mol) in DMF:DMSO with a 4:1 ratio. Afterward, 88.9 µL of CsI solution was added to the second one, to complete the perovskite solution. The solution was spin‐coated first at 1000 rpm for 10 s and then at 6000 rpm for 20 s, both steps with 5000 rpm s^−1^ acceleration rate. After 10 s of the second step, 100 µL of chlorobenzene was released as anti‐solvent on the spinning substrate. The samples were annealed at 100 °C for 1 h in a nitrogen atmosphere.

### Perovskite Fabrication—Cs_0.22_FA_0.78_Pb(I_0.85_Br_0.15_)_3_ + 5% MAPbCl_3_ (see Figure 6)

The 1.68eV perovskite absorber solution was prepared with the precursors FAI, PbI_2_, PbBr_2_, CsI, MACl, PbCl_2_ in DMF and DMSO with the volume ratio of 4:1 and then spin‐coated onto the hole transport layer at 4000rpm for 45 s, 150 µL ethyl acetate was dynamically released as anti‐solvent at 30 sec from the beginning. Samples were annealed at 100 °C for 20 min in N_2_ atmosphere.

### Device Fabrication

The cleaning of glass substrates, the spin‐coated HTL, perovskite film, and PDAI_2_/BAI surface passivation are the same as the half‐stack samples described above. For the electron transport layer (ETL), 20nm C_60_ was thermally evaporated on the perovskite film under a high vacuum of 10^−7^ mbar, then 5nm BCP was thermally evaporated as a hole‐blocking layer. Finally, 100 nm Ag electrode was evaporated under a high vacuum of 10^−7^ mbar.

### Characterization Techniques—Relative Photoluminescence Quantum Yield (rPLQY)‐Imaging

The experimental setup for rPLQY‐imaging consisted of two pulsed 532 nm lasers (SL‐PICOLOYAG‐10, SL‐HGA‐PICOLO‐2, InnoLas Laser), synchronized by a delay generator (DG535, Stanford Research Systems) acted as the master for temporal alignment. The polarization of one of the lasers was rotated by 90° via a lambda half plate (zero‐order half‐wave plate 532 nm, Thorlabs). Subsequently, both lasers were overlaid on a common path by use of a polarizing beamsplitter (laser‐line polarizing beamsplitter 532 nm, Thorlabs). To excite the whole sample the beam diameter was increased using a beam expander unit (compact beam expander 10x @ 532nm, Topag). Detection of the PL signal was done via an industry‐grade complementary metal‐oxide‐semiconductor camera (MC089MG‐SY‐UB, Ximea). It was positioned perpendicular to the sample surface to avoid distortions. The camera acquisition time was set to 100 ms and a 600 nm long pass filter (red filter, 54–762, Edmund Optics) positioned in front of it blocks scattered excitation light (see Figure S3, Supporting Infomation). The camera was placed ≈20 cm away from the sample and, with a 75 mm fixed focal length objective, 7.9 µm spatial resolution was achieved. The camera captured images with a maximum detection window of (1264 × 1264) pixels. All measurements were performed at room temperature to ensure stable PL‐signal detection and consistent temperature dependency of the rate constants (see Figure S4, Supporting Infomation). The initial density was maintained at ≈1.2 × 10^16^ cm^−3^. The delay time for laser pulses ranges from (5–50) ns, depending on the sample, and typically involved image acquisition at 30 different delay times, leading to a total delay time range of 0 ns to (150–1500) ns. To ensure all electrons return to the valence band before each first pulse, a 1 ms window was employed for every double‐pulse excitation. This resulted in 100 double‐pulse excitations per camera acquisition. Considering the above‐mentioned maximum ROI size and acquisition approach, this setup generated roughly 1.6 million data points per run.

### Characterization Techniques—rPLQY‐imaging data processing

The rPLQY‐imaging routine began with a calibration step. This was performed by taking a picture of an unexpanded laser spot with known intensity directed to a white reference. Now the measured signal of the camera with the excitation intensity could be correlated. Then a picture of the expanded beam on the white reference was taken to determine the initial density for each pixel. A Lee‐filter correction was applied to smooth out the speckle pattern. (see Figure S5, Supporting Infomation). Next, the sample was placed on the sample holder and an image in the dark was captured for background correction followed by a PL image at single pulse excitation. Subsequently, double‐pulse excitation was conducted to obtain 30 images at different double‐pulse excitations, with all images being background corrected pixel‐wise by subtracting the dark image. The rPLQY images were obtained by pixel‐wise dividing all double‐pulse images by twice the single‐pulse image according to Equation (2) (see Figure S6, Supporting Infomation). To extract rate constants for the entire sample, the mean values of each rPLQY image were plotted against the corresponding delay time and fitted with Equation S1 (Supporting Infomation). The fitting was performed pixel‐wise to obtain rate‐constant images.

### Characterization Techniques—rPLQY‐Imaging Fitting Routine

The fitting routine employed a curve simulation approach to determine the initial parameter values for the non‐linear equation fitting. This approach helped avoid fitting local minima by simulating varying *k_radiative, ext_* and *k_non‐radiative, eff_* combinations. The simulated curves (see Figure S7, Supporting Infomation) were compared with the actual data and the combination with the least root mean square error (*RMSE*) (see Figure S8, Supporting Infomation) as the initial parameter values were selected. Initially, 1900 curves were simulated for *k_radiative, ext_* ranging from (10^−12^–10^−6^) cm^−3^ s^−1^ and *k_non‐radiative, eff_* from (10^5^–10^11^) s^−1^, with a threshold of *RMSE*< 0.1. Then, the procedure was repeated for the best rate constant combination by adding and subtracting one order of magnitude each, with a stricter threshold of *RMSE*< 0.05 and 9800 simulations. The selected *k_radiative, ext_* and *k_non‐radiative, eff_* combinations were then used as the initial parameter for fitting (see Figure S9, Supporting Infomation). Finally, the extracted rate constants were imaged, and the lifetime for the applied initial density was calculated using τ = *k_non‐radiative, eff_* ^−1^. Data acquisition took less than a minute, while data analysis required ≈20 min with these parameters.

### Characterization Techniques—Power‐Law Parameter k‐Imaging

Two 467 nm LED bars (LDL2‐170/30‐BL2, Creating Customer Satisfation Inc., Puchheim–Germany) were symmetrically aligned for homogeneous sample illumination. The resulting PL signal was recorded by a sCMOS camera (CS2100M‐USB–Quantalux 2.1 MP Monochrome sCMOS Camera, Thorlabs, Bergkirchen–Germany). The maximal detection window of the setup was (135 × 75) mm. The camera was perpendicular positioned 25 cm from the sample. Excitation light was blocked by a 715 nm long pass filter (50.8 mm, SQ 715 nm, LongPass Color Filter, Thorlabs, Bergkirchen–Germany) positioned in front of the camera. Images were acquired at 18 different intensities in the range of 0.01–0.1 suns in 0.005 sun steps. A background correction was performed. The k‐parameter was determined pixel‐wise by plotting the PL signal toward the corresponding excitation intensity *I_in_* and then fitting by PL = *Const*. × *I_in_^k^*.

### Characterization Techniques—Time‐Correlated Single Photon Counting (TCSPC)

TCSPC measurements were conducted on an FLS920 fluorescence spectrometer (Edinburgh Instruments Ltd 2006). A 532 nm laser (SL‐PICOLOYAG‐10, SL‐HGA‐PICOLO‐2, InnoLas Laser) was directed to the sample at a 1 kHz repetition rate and 1.3 ns pulse length. The focused beam radius was 1 mm with excitation intensities of 0.10, 0.24, and 0.61 mW resulting in initial densities of 1.7 × 10^17^ cm^−3^, 4.0 × 10^17^ cm^−3^, and 1.0 × 10^18^ cm^−3^ respectively.

## Conflict of Interest

The authors declare no conflict of interest.

## Supporting information



Supporting Information

## Data Availability

The data that support the findings of this study are available from the corresponding author upon reasonable request.

## References

[1] A. S. R. Bati , Y. L. Zhong , P. L. Burn , M. K. Nazeeruddin , P. E. Shaw , M. Batmunkh , Commun. Mater. 2023, 4, 2.

[2] F. Ma , Y. Zhao , Z. Qu , J. You , Acc. Mater. Res. 2023, 4, 716.

[3] M. A. Green , E. D. Dunlop , M. Yoshita , N. Kopidakis , K. Bothe , G. Siefer , D. Hinken , M. Rauer , J. Hohl‐Ebinger , X. Hao , Prog. Photovoltaics 2024, 32, 425.

[4] NREL , Best Res.‐Cell Efficiencies: Rev 2024, 11.

[5] C. Dreessen , D. Pérez‐del‐Rey , P. P. Boix , H. J. Bolink , J. Lumin. 2020, 222, 117106.

[6] C. M. Wolff , S. A. Bourelle , L. Q. Phuong , J. Kurpiers , S. Feldmann , P. Caprioglio , J. A. Marquez , J. Wolansky , T. Unold , M. Stolterfoht , S. Shoaee , F. Deschler , D. Neher , Adv. Energy Mater. 2021, 11, 2101823.

[7] J. Chen , C. Zhang , X. Liu , L. Peng , J. Lin , X. Chen , Photonics Res. 2021, 9, 151.

[8] H. Cheng , Y. Feng , Y. Fu , Y. Zheng , Y. Shao , Y. Bai , J Mater Chem C Mater 2022, 10, 13590.

[9] T. Kirchartz , J. A. Márquez , M. Stolterfoht , T. Unold , Adv. Energy Mater. 2020, 10, 1904134.

[10] C. Luo , W. Li , D. Xiong , J. Fu , W. Yang , Nanoscale 2019, 11, 15206. 31380885 10.1039/c9nr05217h

[11] B. Hacene , F. Laufer , S. Ternes , A. Farag , R. Pappenberger , P. Fassl , S. Moghadamzadeh , B. A. Nejand , T. Feeney , I. Howard , U. W. Paetzold , Adv. Mater. Technol. 2024, 9, 2301279.

[12] D. B. Sulas , S. Johnston , D. C. Jordan , Sol. Energy Mater. Sol. Cells 2019, 192, 81.

[13] F. Zhang , J. F. Castaneda , T. H. Gfroerer , D. Friedman , Y.‐H. Zhang , M. W. Wanlass , Y. Zhang , Light Sci Appl 2022, 11, 137. 35562347 10.1038/s41377-022-00833-5 PMC9106719

[14] P. Fassl , V. Lami , F. J. Berger , L. M. Falk , J. Zaumseil , B. S. Richards , I. A. Howard , Y. Vaynzof , U. W. Paetzold , Matter 2021, 4, 1391.

[15] H. Shibata , M. Sakai , A. Yamada , K. Matsubara , K. Sakurai , H. Tampo , S. Ishizuka , K.‐K. Kim , S. Niki , Jpn. J. Appl. Phys. 2005, 44, 6113.

[16] T. Schmidt , G. Daniel , K. Lischka , J. Cryst. Growth 1992, 117, 748.

[17] P. Calado , D. Burkitt , J. Yao , J. Troughton , T. M. Watson , M. J. Carnie , A. M. Telford , B. C. O'Regan , J. Nelson , P. R. F. Barnes , Phys. Rev. Appl. 2019, 11, 44005.

[18] M. Kaiser , Y. Li , S. Gharibzadeh , B. S. Richards , U. W. Paetzold , I. A. Howard , Adv. Mater. Technol. 2022, 7, 2200152.

[19] B. A. Ikyo , Am. J. Optic. Photon. 2015, 3, 80.

[20] W. Shockley , W. T. Read , Phys. Rev. 1952, 87, 835.

[21] L. Krückemeier , B. Krogmeier , Z. Liu , U. Rau , T. Kirchartz , Adv. Energy Mater. 2021, 11, 2003489.

[22] J. M. Richter , M. Abdi‐Jalebi , A. Sadhanala , M. Tabachnyk , J. P. H. Rivett , L. M. Pazos‐Outón , K. C. Gödel , M. Price , F. Deschler , R. H. Friend , Nat. Commun. 2016, 7, 13941. 28008917 10.1038/ncomms13941 PMC5196482

[23] F. Staub , T. Kirchartz , K. Bittkau , U. Rau , J. Phys. Chem. Lett. 2017, 8, 5084. 28976758 10.1021/acs.jpclett.7b02224

[24] M. Kaiser , Y. Li , I. Allegro , B. S. Richards , U. W. Paetzold , I. A. Howard , Adv. Mater. Interfaces 2021, 8, 2101326.

[25] J. C. de Mello , H. F. Wittmann , R. H. Friend , Adv. Mater. 1997, 9, 230.

[26] Y. Li , I. Allegro , M. Kaiser , A. J. Malla , B. S. Richards , U. Lemmer , U. W. Paetzold , I. A. Howard , Mater. Today 2021, 49, 35.

[27] T. W. Crothers , R. L. Milot , J. B. Patel , E. S. Parrott , J. Schlipf , P. Müller‐Buschbaum , M. B. Johnston , L. M. Herz , Nano Lett. 2017, 17, 5782. 28792767 10.1021/acs.nanolett.7b02834

[28] Y. Yamada , T. Nakamura , M. Endo , A. Wakamiya , Y. Kanemitsu , J. Am. Chem. Soc. 2014, 136, 11610. 25075458 10.1021/ja506624n

[29] M. A. Ruiz‐Preciado , F. Gota , P. Fassl , I. M. Hossain , R. Singh , F. Laufer , F. Schackmar , T. Feeney , A. Farag , I. Allegro , H. Hu , S. Gharibzadeh , B. A. Nejand , V. S. Gevaerts , M. Simor , P. J. Bolt , U. W. Paetzold , ACS Energy Lett. 2022, 7, 2273. 35844471 10.1021/acsenergylett.2c00707 PMC9274764

[30] A. Ren , H. Lai , X. Hao , Z. Tang , H. Xu , B. M. F. Yu Jeco , K. Watanabe , L. Wu , J. Zhang , M. Sugiyama , J. Wu , D. Zhao , Joule 2020, 4, 1263.

[31] R. G. Sargent , In *Proceedings of the 2010 Winter Simulation Conf*., Baltimore, MD, USA, December, 2010, pp. 166–183.

[32] J. Qin , X.‐K. Liu , C. Yin , F. Gao , Trends Chem 2021, 3, 34.

[33] B. Krogmeier , F. Staub , D. Grabowski , U. Rau , T. Kirchartz , Sustainable Energy Fuels 2018, 2, 1027.

[34] Y. Wang , S. Akel , B. Klingebiel , T. Kirchartz , Adv. Energy Mater. 2024, 14, 2302614.

[35] E. M. Hutter , J.‐J. Hofman , M. L. Petrus , M. Moes , R. D. Abellón , P. Docampo , T. J. Savenije , Adv. Energy Mater. 2017, 7, 1602349.

[36] Z. Liu , L. Krückemeier , B. Krogmeier , B. Klingebiel , J. A. Márquez , S. Levcenko , S. Öz , S. Mathur , U. Rau , T. Unold , T. Kirchartz , ACS Energy Lett. 2019, 4, 110.

[37] W. Yan , L. Mao , P. Zhao , A. Mertens , S. Dottermusch , H. Hu , Z. Jin , B. S. Richards , Opt. Express 2020, 28, 15706. 32403592 10.1364/OE.392246

[38] L. M. Herz , Annu. Rev. Phys. Chem. 2016, 67, 65. 26980309 10.1146/annurev-physchem-040215-112222

[39] M. B. Johnston , L. M. Herz , Acc. Chem. Res. 2016, 49, 146. 26653572 10.1021/acs.accounts.5b00411

[40] J. Roger , L. K. Schorn , M. Heydarian , A. Farag , T. Feeney , D. Baumann , H. Hu , F. Laufer , W. Duan , K. Ding , A. Lambertz , P. Fassl , M. Worgull , U. W. Paetzold , Adv. Energy Mater. 2022, 12, 2200961.

[41] L. Shi , M. Zhang , Y. Cho , T. L. Young , D. Wang , H. Yi , J. Kim , S. Huang , A. W. Y. Ho‐Baillie , ACS Appl. Energy Mater. 2019, 2, 2358.

[42] S. P. Dunfield , D. T. Moore , T. R. Klein , D. M. Fabian , J. A. Christians , A. G. Dixon , B. Dou , S. Ardo , M. C. Beard , S. E. Shaheen , J. J. Berry , M. F. A. M. van Hest , ACS Energy Lett. 2018, 3, 1192.

[43] U. Rau , U. W. Paetzold , T. Kirchartz , Phys. Rev. B 2014, 90, 35211.

[44] C. Ulbrich , M. Peters , B. Bläsi , T. Kirchartz , A. Gerber , U. Rau , Opt. Express 2010, 18, A133. 20588581 10.1364/OE.18.00A133

[45] M. Schultes , T. Helder , E. Ahlswede , M. F. Aygüler , P. Jackson , S. Paetel , J. A. Schwenzer , I. M. Hossain , U. W. Paetzold , M. Powalla , ACS Appl. Energy Mater. 2019, 2, 7823.

[46] T. Feeney , I. M. Hossain , S. Gharibzadeh , F. Gota , R. Singh , P. Fassl , A. Mertens , A. Farag , J.‐P. Becker , S. Paetel , E. Ahlswede , U. W. Paetzold , Sol. RRL 2022, 6, 2200662.

[47] M. Stolterfoht , C. M. Wolff , J. A. Márquez , S. Zhang , C. J. Hages , D. Rothhardt , S. Albrecht , P. L. Burn , P. Meredith , T. Unold , D. Neher , Nat. Energy 2018, 3, 847.

[48] C. M. Wolff , P. Caprioglio , M. Stolterfoht , D. Neher , Adv. Mater. 2019, 31, 1902762. 10.1002/adma.201902762 31631441

[49] J. Warby , F. Zu , S. Zeiske , E. Gutierrez‐Partida , L. Frohloff , S. Kahmann , K. Frohna , E. Mosconi , E. Radicchi , F. Lang , S. Shah , F. Peña‐Camargo , H. Hempel , T. Unold , N. Koch , A. Armin , F. De Angelis , S. D. Stranks , D. Neher , M. Stolterfoht , Adv. Energy Mater. 2022, 12, 2103567.

[50] D. Menzel , A. Al‐Ashouri , A. Tejada , I. Levine , J. A. Guerra , B. Rech , S. Albrecht , L. Korte , Adv. Energy Mater. 2022, 12, 2201109. 10.1021/acsami.1c10171 34472345

[51] M. Stolterfoht , P. Caprioglio , C. M. Wolff , J. A. Márquez , J. Nordmann , S. Zhang , D. Rothhardt , U. Hörmann , Y. Amir , A. Redinger , L. Kegelmann , F. Zu , S. Albrecht , N. Koch , T. Kirchartz , M. Saliba , T. Unold , D. Neher , Energy Environ. Sci. 2019, 12, 2778.

[52] S.‐G. Choi , S.‐K. Jung , J.‐H. Lee , J.‐H. Kim , W. Zheng , J.‐W. Lee , ACS Energy Lett. 2024, 9, 5360.

[53] L. Canil , T. Cramer , B. Fraboni , D. Ricciarelli , D. Meggiolaro , A. Singh , M. Liu , M. Rusu , C. M. Wolff , N. Phung , Q. Wang , D. Neher , T. Unold , P. Vivo , A. Gagliardi , F. De Angelis , A. Abate , Energy Environ. Sci. 2021, 14, 1429.

[54] M. G. Abebe , A. Abass , G. Gomard , L. Zschiedrich , U. Lemmer , B. S. Richards , C. Rockstuhl , U. W. Paetzold , Phys. Rev. B 2018, 98, 75141.

[55] S. McDowall , T. Butler , E. Bain , K. Scharnhorst , D. Patrick , Appl. Opt. 2013, 52, 1230. 23434994 10.1364/AO.52.001230

[56] V. Sittinger , P. S. C. Schulze , C. Messmer , A. Pflug , J. C. Goldschmidt , Opt. Express 2022, 30, 37957. 36258374 10.1364/OE.458953

[57] C. Cho , B. Zhao , G. D. Tainter , J.‐Y. Lee , R. H. Friend , D. Di , F. Deschler , N. C. Greenham , Nat. Commun. 2020, 11, 611. 32001711 10.1038/s41467-020-14401-1 PMC6992794

