# Supplementary material for: Imaging of Recombination Rates and Lifetime in Perovskite Thin Film Processing
Source: Small Methods. 2025 Feb 16;9(7):2402119. doi: 10.1002/smtd.202402119 (PMC12285638; doi:10.1002/smtd.202402119)
Supplement: Supplementary file 1 — Supporting Information [file SMTD-9-2402119-s001.pdf]

small
methods

Supporting Information

for *Small Methods*, DOI 10.1002/smtd.202402119

Imaging of Recombination Rates and Lifetime in Perovskite Thin Film Processing

*Benjamin Hacene, Nils W. Rosemann, Julie Roger, Xuzheng Liu, Daniel O. Baumann, Ronja Pappenberger, Mohammad Gholipoor, Hannah Racky, Paul Fassl, Ian A. Howard and Ulrich W. Paetzold**

Supporting Information (SI)

Imaging of Recombination Rates and Lifetime in Perovskite Thin Film Processing

*Benjamin Hacene[1], Nils W. Rosemann[1], Julie Roger[2], Xuzheng Liu[1], Daniel O. Baumann[2], Ronja Pappenberger[2], Mohammad Gholipoor[2], Hannah Racky[1], Paul Fassl[2], Ian A. Howard[1,2], and Ulrich W. Paetzold[1, 2, *]*

B. Hacene, N. W. Rosemann, X. Liu, H. Racky, I. A. Howard, U. W. Paetzold
[1] Light Technology Institute (LTI), Karlsruhe Institute of Technology (KIT), Engesserstrasse 13, 76131 Karlsruhe, Germany

J. Roger, D. Baumann, R. Pappenberger, M. Gholipoor, P. Fassl, I. A. Howard, U. W. Paetzold
[2] Institute of Microstructure Technology (IMT), Karlsruhe Institute of Technology (KIT), Hermann-von-Helmholtz-Platz 1, 76344 Eggenstein-Leopoldshafen, Germany

* corresponding author contact details: ulrich.paetzold@kit.edu

Table of Content

Equations for simulations and fits ……….. 2
S1 & S2 2

Figures …………………………………… 3
S1 & S2 3
S3 & S4 4
S4 & S6 5
S7 & S8 6
S9 7

References ………………………………... 7

Equations for simulations and fits

The model proposed by Kaiser *et al.*[1] considers exciton and free carrier recombination in two-dimensional (2D) and three-dimensional (3D) perovskites. Given that this investigation is focused on 3D materials, which are mainly associated with free carrier recombination[2], the analysis refers to the free carrier recombination component of the model.

The relative photoluminescence quantum yield (rPLQY) is fitted with the following equation:

$$\mathrm{rPLQY}(t_{delay}) = \frac{\mathrm{PLQY}(t_{delay})}{\mathrm{PLQY}(t_{delay} \to \infty)} =$$

$$\frac{\left(\frac{-\left(\exp(k_{nr}t_{delay})\left(\frac{{k_{nr}}^2}{k_r} + N_{0,A}k_{nr}\right) - t_{delay}\exp(k_{nr}t_{delay})\left(\frac{{k_{nr}}^3}{k_r} + N_{0,A}{k_{nr}}^2\right) + N_{0,A}{k_{nr}}^2 t_{delay}\right) \times k_r}{\left(k_{nr}\exp(k_{nr}t_{delay}) - N_{0,A}k_r + N_{0,A}k_r\exp(k_{nr}t_{delay})\right)} - \left(k_{nr}\ln\left(k_{nr}\exp(k_{nr}t_{delay}) - N_{0,A}k_r + N_{0,A}k_r\exp(k_{nr}t_{delay})\right)\right) + \left(k_{nr} + N_{0,A}k_r + k_{nr}\ln(k_{nr})\right) + \left(k_r N_1 + k_{nr}\ln\left(\frac{k_{nr}}{k_{nr} + k_r N_1}\right)\right)\right)}{\left(k_r\, N_{0,A} + k_{nr}\ln\left(\frac{k_{nr}}{k_{nr} + k_r N_{0,A}}\right)\right) + \left(k_r\, N_{0,B} + k_{nr}\ln\left(\frac{k_{nr}}{k_{nr} + k_r N_{0,B}}\right)\right)}$$

with

$$N_1 = N_{0,B} + n(t_{delay}) = N_{0,B} + \frac{k_{nr}}{k_r\left(\exp(k_{nr}t_{delay}) - 1\right) + \frac{k_{nr}}{N_{0,A}}\exp(k_{nr}t_{delay})} \qquad \text{(S1)}$$

with the external radiative and effective non-radiative rate constant k_r and k_{nr} respectively. The initial densities of the first and second laser pulse $N_{0,A}$ and $N_{0,B}$ and the delay time t_{delay}. N_1 describes the density after the second pulse.

The double-pulse induced PL decay is described as follows:

$$\mathrm{PL}(t) \propto k_r n'^2(t) =$$

$$\begin{cases} k_r\left(\frac{\mathrm{k_{nr}}}{k_r(\exp(k_{nr}t) - 1) + \frac{k_{nr}}{N_{0,A}}\exp(k_{nr}t)}\right)^2 ; \; t < t_{delay} \\ k_r\left(\frac{k_{nr}}{k_r\left(\exp\left(k_{nr}(t - t_{delay})\right) - 1\right) + \frac{k_{nr}}{N_1}\exp\left(k_{nr}(t - t_{delay})\right)}\right)^2 ; \; t \geq t_{delay} \end{cases} \qquad \text{(S2)}$$

Kaiser *et al.*[1] provides a detailed derivation of the equations S1 and S2.

Figures

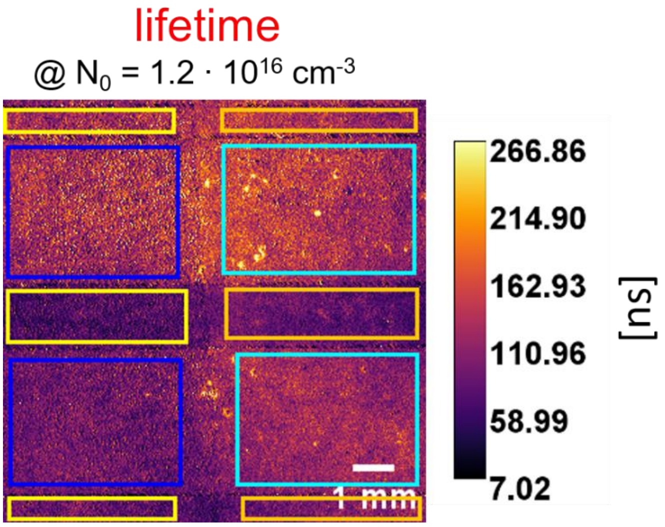

Figure S1: Lifetime image of glass / ITO / 2PACz / perovskite sample treated on the lefthand side with heat and on the righthand side with heat and pressure. The dark blue and cyan ROIs indicate areas with ITO. The yellow and orange ROIs indicate areas without ITO.

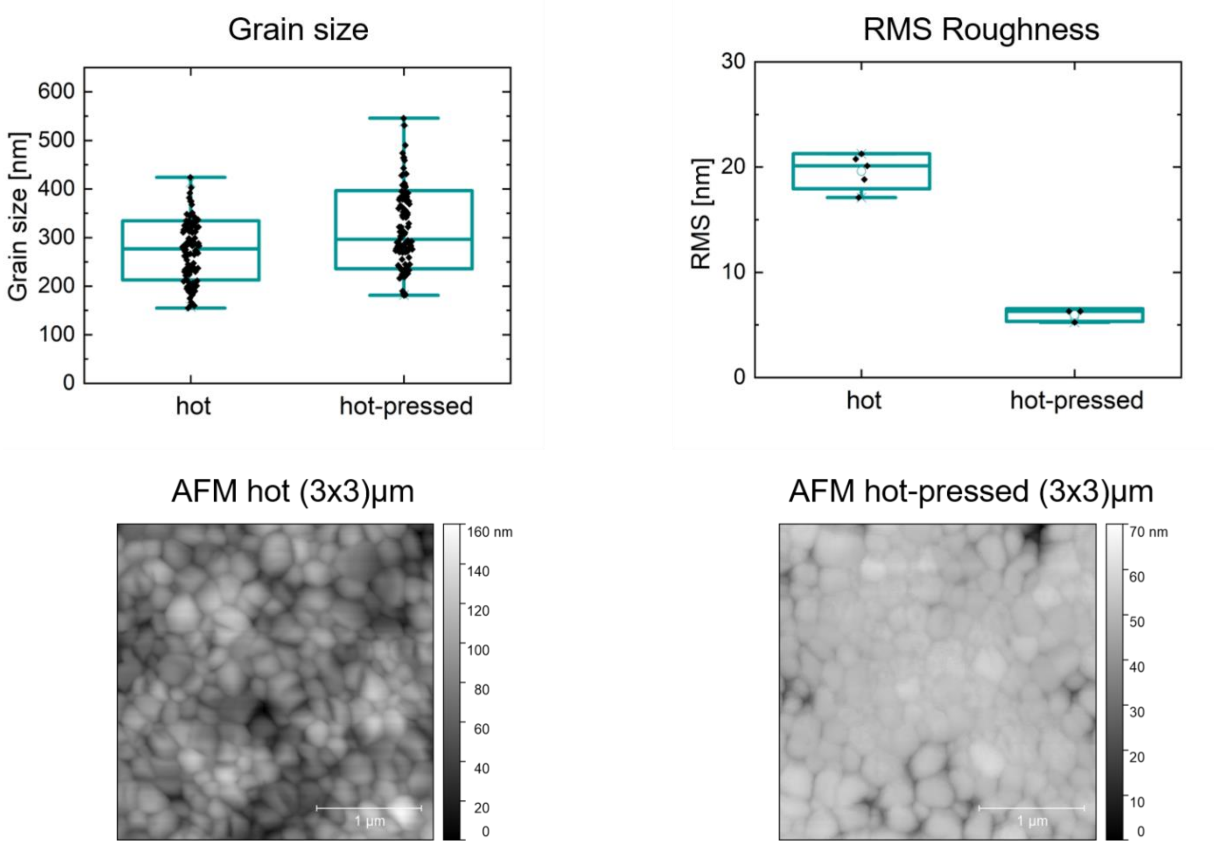

Figure S2: Grain size and roughness comparison conducted by atomic force microscopy (AFM) of glass / ITO / 2PACz / perovskite sample treated on the lefthand side with heat and on the righthand side with heat and pressure.

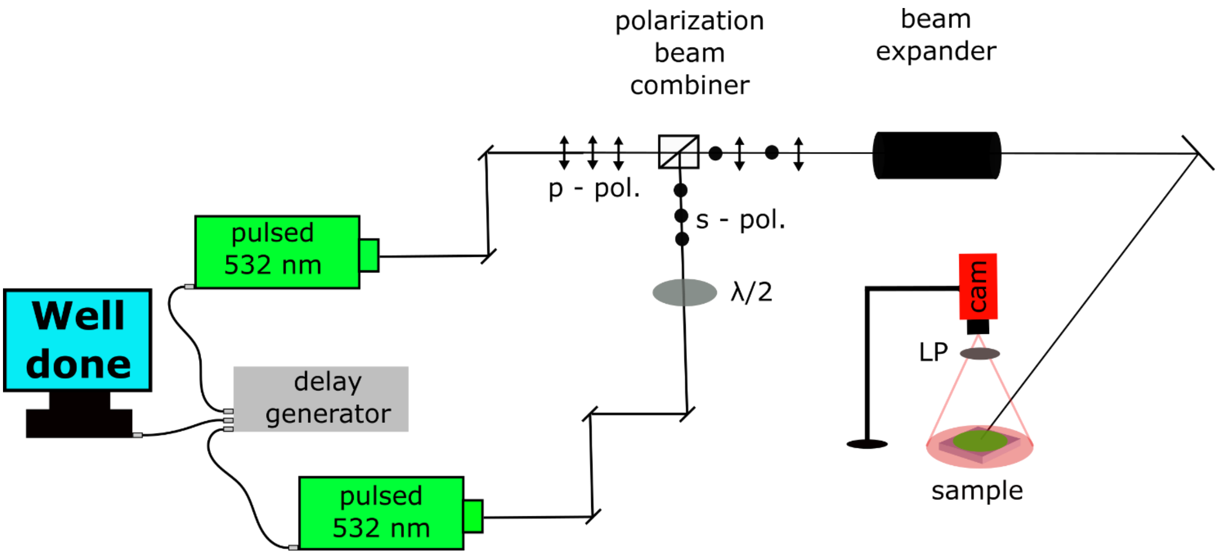

Figure S3: Schematic illustration of setup.

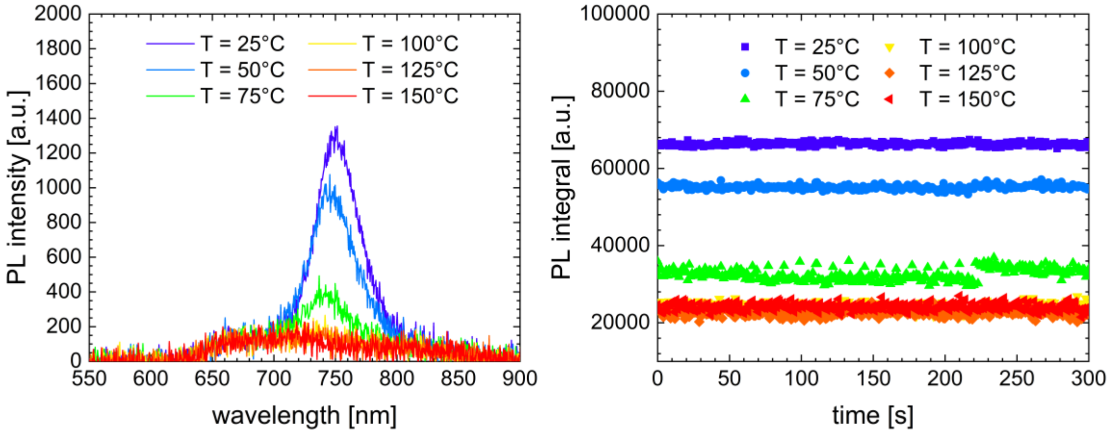

Figure S4: On the lefthand side PL spectra at different temperatures of a $Cs_{0.1}(MA_{0.17}FA_{0.83})_{0.9}Pb(I_{0.83}Br_{0.17})_3$ perovskite thin film are shown. A reasonable signal to noise ratio up to 75°C is visible. On the righthand side the PL integral of each spectrum is shown over time.

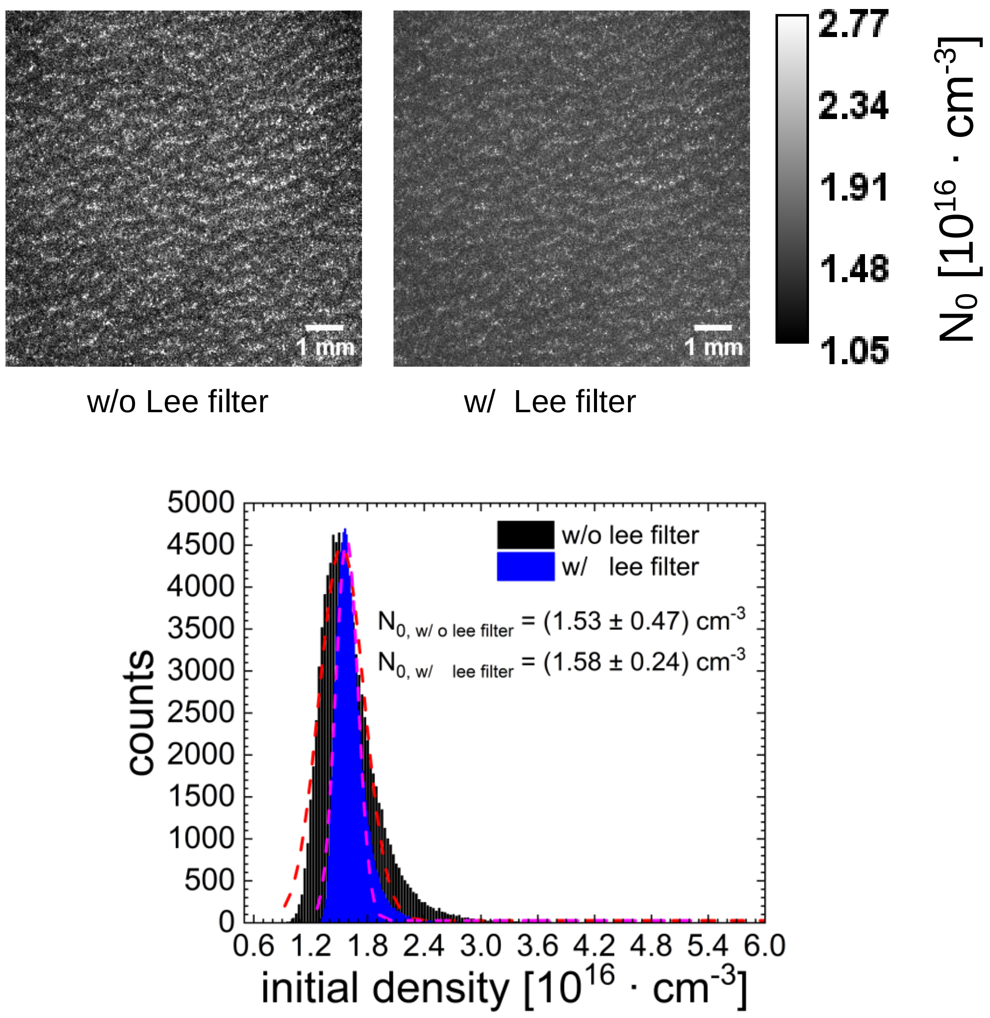

Figure S5: Initial density image and distribution w/o and w/ Lee filter

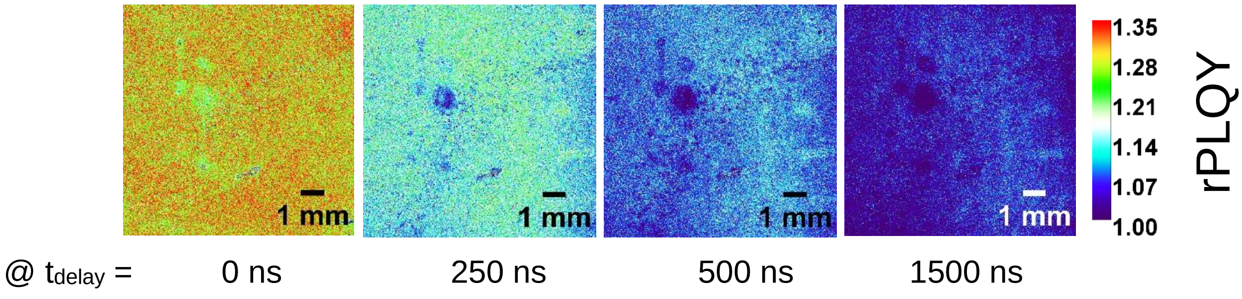

Figure S6: Exemplary rPLQY – images at 0 ns, 250 ns, 500 ns, 1500 ns delay time.

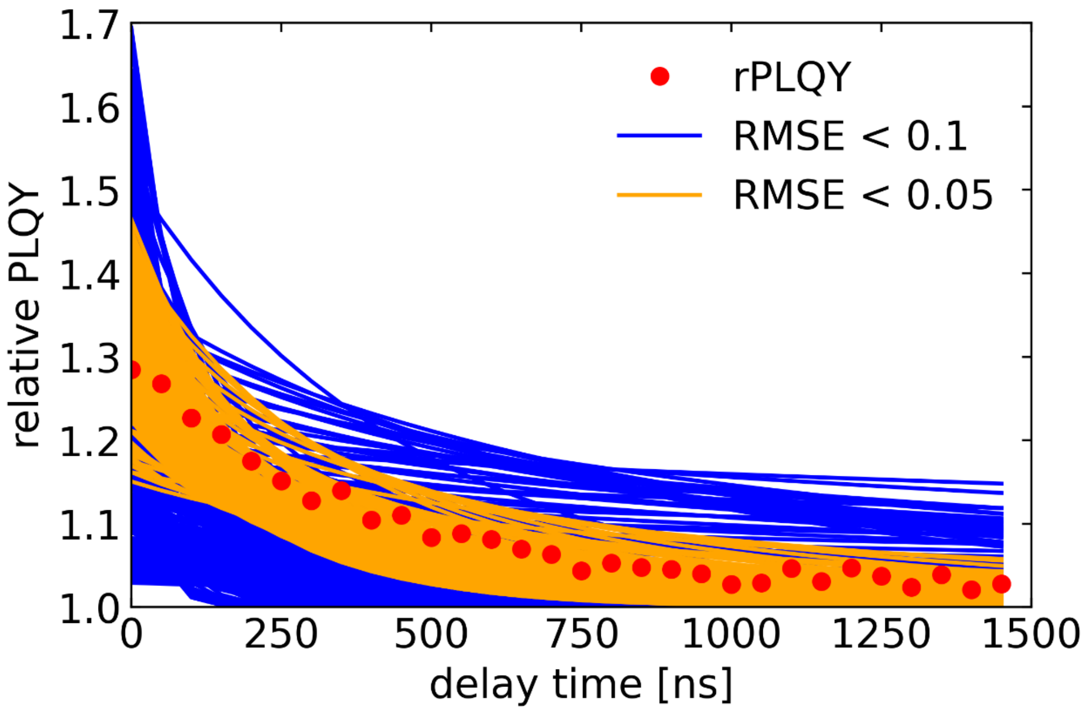

Figure S7: Exemplary simulated rPLQY curves with root meen square errors (*RMSE*) < 0.1 and 0.05.

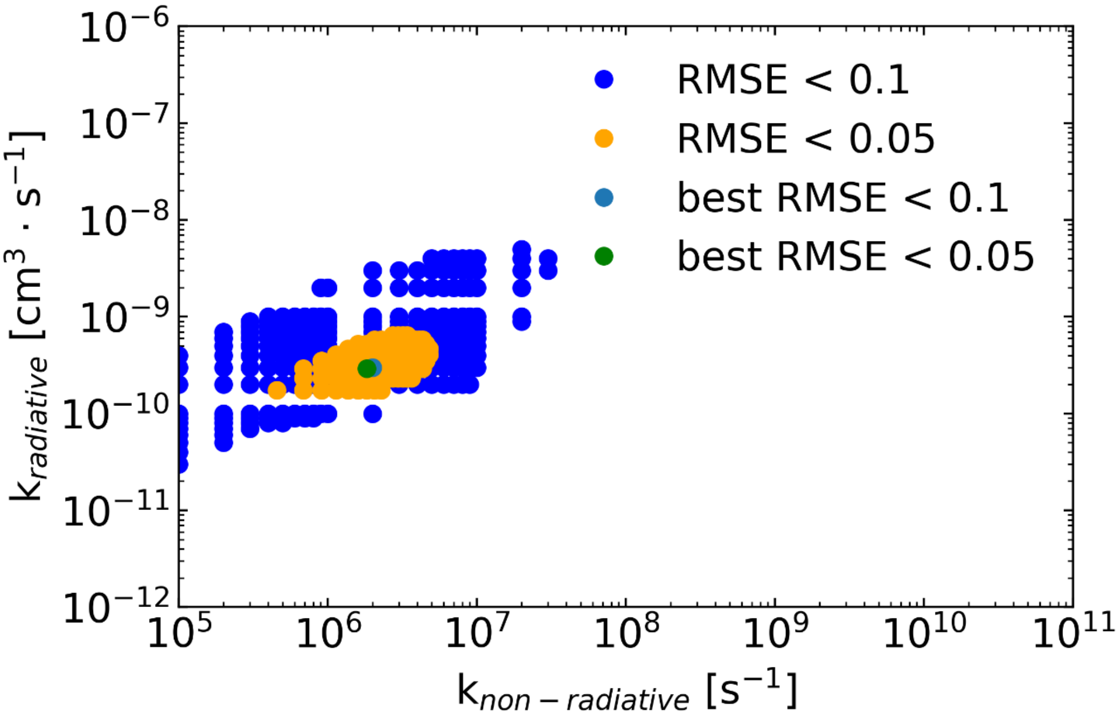

Figure S8: External radiative recombination rate constant is simulated in a range of (10^{-12} – 10^{-6}) $cm^{-3} \cdot s^{-1}$ and effective non-radiative recombination rate constant in a range of (10^{5} – 10^{11}) s^{-1}. Blue and orange areas indicate rate constant combinations leading to rPLQY curves with *RMSE* < 0.1 and 0.05.

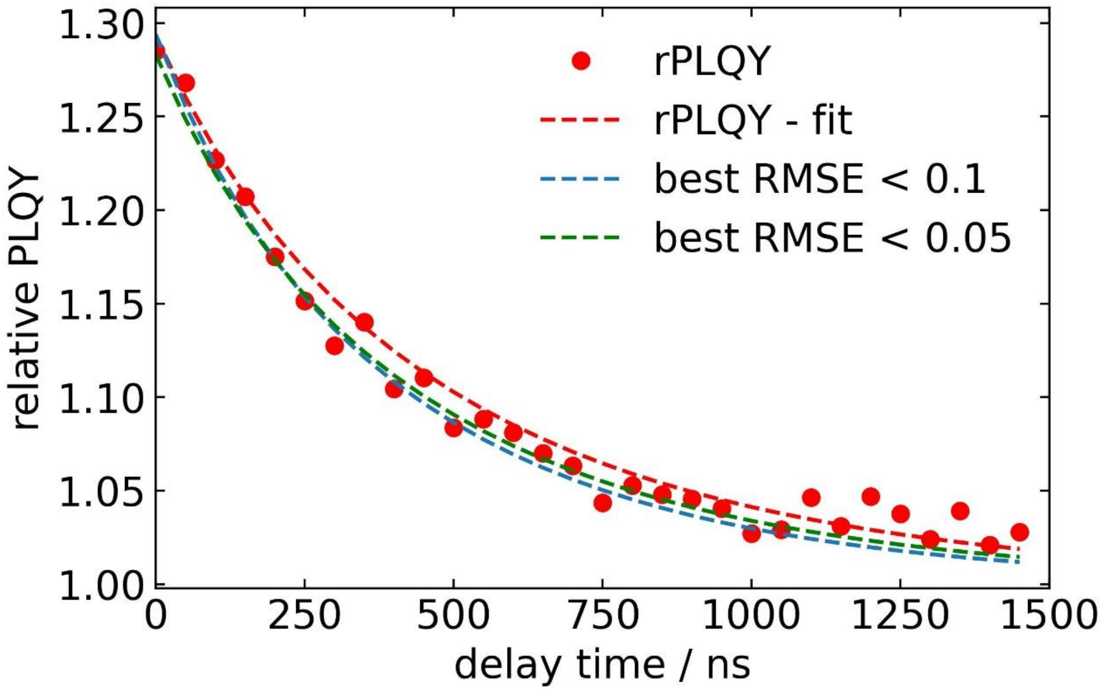

Figure S9: Exemplary rPLQY data, best fit and best simulated curve for *RMSE* < 0.1 and <0.05.

References

[1] M. Kaiser, Y. Li, S. Gharibzadeh, B. S. Richards, U. W. Paetzold, I. A. Howard, *Adv Mater Technol* **2022**, *7*, 2200152.

[2] Y. Li, I. Allegro, M. Kaiser, A. J. Malla, B. S. Richards, U. Lemmer, U. W. Paetzold, I. A. Howard, *Materials Today* **2021**, *49*, 35.
